# Comprehensive whole genome sequence analyses yields novel genetic and structural insights for Intellectual Disability

**DOI:** 10.1186/s12864-017-3671-0

**Published:** 2017-05-24

**Authors:** Farah R. Zahir, Jill C. Mwenifumbo, Hye-Jung E. Chun, Emilia L. Lim, Clara D. M. Van Karnebeek, Madeline Couse, Karen L. Mungall, Leora Lee, Nancy Makela, Linlea Armstrong, Cornelius F. Boerkoel, Sylvie L. Langlois, Barbara M. McGillivray, Steven J. M. Jones, Jan M. Friedman, Marco A. Marra

**Affiliations:** 1 Canada’s Michael Smith Genome Sciences Center, Vancouver, BC V5Z 4S6 Canada; 2 Department of Medical Genetics, University of British Columbia, Vancouver, BC V6T 1Z4 Canada; 3 Department of Pediatrics, Centre for Molecular Medicine & Therapeutics Child & Family Research Institute, University of British Columbia, Vancouver, BC V6T 1Z4 Canada; 4 Provincial Medical Genetics Programme, Children’s & Women’s Health Centre of British Columbia, Vancouver, BC V6H 3N1 Canada; 5 Qatar Biomedical Research Institute, Hamad Bin Khalifa University, P.O. Box 34110, Doha, Qatar

**Keywords:** Intellectual Disability, Whole genome sequencing, *ARID1B*, *PHF6*, *SPRY4*, *CACNB3*, *SQSTM1*, *UPF1*, 1q43 microdeletion, Genome assembly

## Abstract

**Background:**

Intellectual Disability (ID) is among the most common global disorders, yet etiology is unknown in ~30% of patients despite clinical assessment. Whole genome sequencing (WGS) is able to interrogate the entire genome, providing potential to diagnose idiopathic patients.

**Methods:**

We conducted WGS on eight children with idiopathic ID and brain structural defects, and their normal parents; carrying out an extensive data analyses, using standard and discovery approaches.

**Results:**

We verified *de novo* pathogenic single nucleotide variants (SNV) in *ARID1B c.1595delG* and *PHF6 c.820C > T*, potentially causative *de novo* two base indels in *SQSTM1 c.115_116delinsTA* and *UPF1 c.1576_1577delinsA,* and *de novo* SNVs in *CACNB3 c.1289G > A,* and *SPRY4 c.508 T > A,* of uncertain significance. We report results from a large secondary control study of 2081 exomes probing the pathogenicity of the above genes. We analyzed structural variation by four different algorithms including *de novo* genome assembly. We confirmed a likely contributory 165 kb *de novo* heterozygous 1q43 microdeletion missed by clinical microarray. The *de novo* assembly resulted in unmasking hidden genome instability that was missed by standard re-alignment based algorithms. We also interrogated regulatory sequence variation for known and hypothesized ID genes and present useful strategies for WGS data analyses for non-coding variation.

**Conclusion:**

This study provides an extensive analysis of WGS in the context of ID, providing genetic and structural insights into ID and yielding diagnoses.

**Electronic supplementary material:**

The online version of this article (doi:10.1186/s12864-017-3671-0) contains supplementary material, which is available to authorized users.

## Background

Intellectual Disability (ID) affects 1–3% of the global population. A significant proportion of ID is caused by genetic defects, yet despite extensive testing including by clinical chromosomal microarray (CMA), ~30% of cases remain idiopathic [1].

Genome-wide sequencing can identify previously unknown genes causative for ID. Whole exome sequencing (WES) is limited by poor ability or inability to detect non-coding and structural variation, and capturing less than 100% of the exome [2]. In contrast, whole genome sequencing (WGS) offers a comprehensive screen of a variety of DNA variation types. Current evidence suggests WGS is able to detect coding variants in 42% of cases missed by WES [2].

**Fig. 1:**
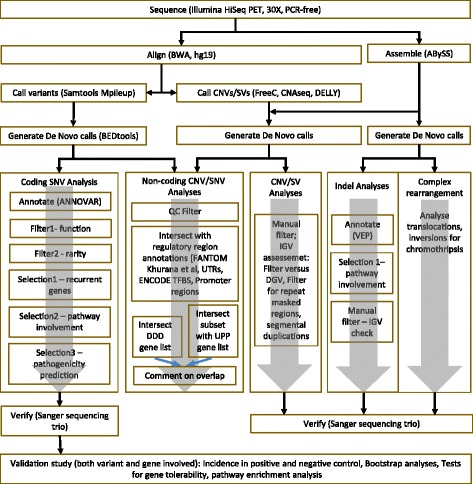
Schematic of complete study design. Abbreviations: CNV = copy number variant; SV = structural variant; SNV = single nucleotide variant; DDD = Deciphering Developmental Disabilities study; UPP = Ubiquitin Proteolysis Pathway; IGV = Integrated Genome Viewer; DGV = Database of Genome Variation

We report comprehensive WGS analyses for eight patients with ID and brain malformations, whose family history suggested a *de novo* mutation. Despite a diagnostic odyssey, including genome-wide clinical and research CMA, they were idiopathic. WGS was conducted on trios composed of the affected child and both unaffected parents (average 34X coverage), and data was analyzed using both alignment and assembly approaches to detect all possible causative genetic changes- single nucleotide variants (SNVs and indels), copy number variants (CNVs) and structural variants (SVs) (Fig. 1). We validated our findings using WES data from an independent positive control cohort of 2081 patients with ID and other neurocognitive phenotypes, and a negative control WGS cohort of 2535 normal subjects. In addition we probed molecular themes indicated by our discovery cohort findings in the positive control cohort, leveraging its large size. We also conducted a screen for *de novo* variants in possible regulatory sequences of known and hypothesized pathogenic genes.

## Methods

### Subjects

Patients were enrolled from the British Columbia Children’s and Women’s Hospital Provincial Medical Genetics Program after obtaining informed consent. This study is approved by the British Columbia Children’s and Women’s hospital research ethics boards. All patients presented with ID (moderate to severe) and brain morphological defects detected by MRI or CT scan. Patients had no family history of ID, and all were products of normal pregnancies with no reported teratogenic exposures as ascertained by clinical assessment by board certified Medical Genetics specialists at the recruiting facility. Saliva samples were collected and DNA extracted using DNA Genotek® collection kits, reagents and protocols from child, father and mother.

### Methods

WGS methods, variant calling protocols, verification methods, and secondary control study methods including bootstrap analysis, are summarized below and detailed in Additional file 1. Briefly; DNA was extracted using DNAGenotek® extraction kits. Paired-end WGS libraries were prepared using Illumina’s PCR-free protocol (TruSeq DNA Sample prep kit -Illumina Catalogue Number FC-121-1002). Sequencing was by IlluminaHiSeq 2500 platform (v3 chemistry) generating 100 bp paired-end reads, using three lanes per sample (34X average coverage across all samples). Alignment and variant calling was by Canada’s Michael Smith Genome Science Center standard pipelines (Additional file 1, reference genome - hg19).

Variants were identified and filtered as follows, briefly; putative SNVs were identified using SAMtools mpileup version 0.1.17 run on each sample separately. Relatedness was tested for each trio by comparing SNP concordance between child, mother and father using vcftools-0.1.14 [3] (Additional file 2: Table S1). *De novo* variants were selected by intersecting the child’s SNVs with that of each parent, and selecting variants only present in the child and not in either parent. For variants in the coding region, we selected *de novo* missense, nonsense and splicing variants, i.e., functional variants. We next selected rare variants by excluding alleles with minor allele frequency >1% in dbSNPv135 (excluding disease associated variants), Exome Variant Server, Exome Aggregation Consortium (ExAC), and in-house databases of >7430 exomes, and >3000 genomes (at Canada’s Michael Smith Genome Sciences Center and the British Columbia Children’s Hospital Research Center, available via open-source access [4]). We then used pathway enrichment analyses to prioritize *de novo* rare variants; selecting SNVs in genes enriched in pathways involved in brain development and function conducted using QIAGENs Ingenuity® Pathway Analysis (IPA), DAVID (https://david-d.ncifcrf.gov/ 6.7) and Panther (http://pantherdb.org/). For those variants passing the pathway enrichment screen, pathogenicity predictions and conservation scores were annotated using SIFT [5], PhyloP [6], PolyPhen [7], MutationTaster [8] and CADD [9] scores. These steps yielded *de novo*, functional, rare variants, that are highly conserved and predicted to be damaging and in biologically relevant pathways. In addition to the above prioritization, the rare functional variants were subsequently also screened under a series of additional genetic models (e.g. compound heterozygous, *de novo* heterozygous, homozygous recessive, hemizygous recessive), and manually checked for alignment quality with Integrated Genomic Viewer (IGV, https://www.broadinstitute.org/software/igv). SNVs that were highly conserved and were predicted to be damaging by at least one pathogenicity prediction software, were selected for verification by Sanger sequencing in the child, mother and father.

CNV analyses was conducted using FREEC [10], CNAseq [11], DELLY [12] and ABySS [13]. The first three algorithms align reads to the reference genome while ABySS uses *de novo* assembly to reconstruct the patient’s genome. SV analyses was conducted using only DELLY and ABySS. First, *de novo* CNVs/SVs were identified by comparing the child’s data to that of either parent (Additional file 1). *De novo* CNVs from each algorithm were filtered by manual assessment of local read configuration on IGV, and genuine ones were prioritized based on functional relevance of the included/CNV-affected genes. SVs, i.e., translocations and inversions, were filtered by either IGV read visualization and then by using QC metrics specific to each algorithm; QC metrics generated by the program were used for DELLY, and checking of BLAT scores for breakpoint-junction contigs and number of supporting reads were used for ABySS. Candidate CNVs/SVs that were selected from the above filtering were verified using an independent method as detailed below.

All *de novo* variants, i.e., SNVs, CNVs and SVs, were verified by Sanger sequencing of PCR-captured amplicons of the affected sequence, either bearing the SNV or spanning the breakpoint junction (in the case of CNVs and SVs) in the trio, with forward and reverse primers (Additional file 3: Table S2). All verified candidate SNVs were subjected to genotype-phenotype correlations assessment as per the guidelines of the American College of Medical Genetics (ACMG) [14].

Secondary control study - WES data from the UK10K project [15] for 2081 patients with neurofunctional phenotypes (available clinical data for the projects that comprise this cohort is found in Additional file 4: Table S3), and WGS data from 2535 normal individuals from the 1000 Genomes project [16]; a publicly available repository of variation in healthy individuals, was obtained. ‘Possibly damaging SNVs’ (PDSs), were extracted from these datasets (as detailed in Additional file 1 and Additional file 5: Figure S2), and a gene-wise PDS burden for all genes in the human genome was determined in both the positive and negative control cohorts. Subsequently the gene-wise PDS burden only in our candidate genes was compared between the positive and negative control cohorts. We further bootstrapped the positive control cohort to determine if the PDS burden in our six candidate genes could be due to random sampling. Finally, we tested to see what functional pathways genes with PDS in the positive control cohort were involved in, and conducted a Kyoto Encyclopedia of Genes and Genomes (KEGG [17]) pathway enrichment analyses, testing which of the total 57 functional pathways from KEGG were most enriched for genes bearing PDS in this large dataset.

Regulatory region variation – For our regulatory region analysis we selected ‘high confidence’ *de novo* SNVs defined as having a mapping quality > 30 and read depth ≥ 10 and ≤ 100, and ‘high confidence’ *de novo* CNVs defined as those that were detected by two or more CNV detection algorithms. We then intersected both the *de novo* high confidence SNVs and CNVs with six non-coding sequence annotation datasets. Results from the above, i.e., *de novo* high confidence SNVs and CNVs with involvement in putative regulatory regions, were then intersected with candidate gene lists and appropriate flanking sequence (Additional file 1) to determine their possible association to a candidate known or hypothesized ID gene.

## Results

### *De novo* SNVs identified by objective molecular pathway-based filtration

Genes with functional rare *de novo* SNVs were screened using three pathway analyses programs (IPA, DAVID and Panther) in order to refine candidates involved in brain development and function; IPA returned 17 candidate genes, DAVID returned 23, and Panther returned 9. A total of 23 unique genes involved in brain development and function were yielded by the combined analyses (i.e., found by at least one of the programs). From these, highly conserved and predicted damaging SNVs (11 SNVs in 11 genes in six patients) were Sanger tested, and six SNVs in six genes in five children were confirmed as heterozygous *de novo* (Table 1); *ARID1B* [MIM:614556] NM_017519:c.1595delG (p.G532fs), *PHF6*[MIM:300414] NM_001015877:c.820C > T (p.R274X), *SPRY4* [MIM:607984] NM_001127496:c.508 T > A (p.C170S)*, CACNB3*[MIM:601958] NM_0012069:c.1289G > A (p.R430Q)*, SQSTM1* [MIM:601530] NM_03900: c.115_116delinsTA (p.A39fr*#) and *UPF1* [MIM:601430] NM_002911:c.1576_1577delinsAA (p.A526N). The latter two were found in a single patient while the rest each appeared in a separate patient. As best practice, we also screened our *de novo* rare functional variants for location within published known [2] and candidate ID genes [18], however no new findings were yielded. Except *ARID1B* and *PHF6*, the other genes are novel for ID. Table 1 provides variant classification as per the ACMG variant interpretation guidelines [14] (Additional file 6: Table S4 for detailed classification of variants) and our interpretation of their causative effect. Brief genotype-phenotype correlations are given below;

**Table 1:** Patient phenotype and variant summary

Patient #	Approx Age at Examination^a^	Phenotype	Gene	Exonic function	AAChange	Chr	Co-ordinate (Hg19)	Other info	ACMG classification system	Comment	Other reports of same variant
43	Less than 5 years old	Feeding problems and failure to thrive, global developmental delay, Autism. Height 25%ile, weight -3SD, OFC 2-10%ile. CT/MRI-Dysgenesis of the corpus callosum.	*ARID1B*	Frame-shift single base deletion	NM_017519:c.1595delG:p.G532fs	6	157150413	het	PVS1, PS2, PM2 = Pathogenic	Sufficient to cause disease	
58	Less than 5 years old	Developmental delay. Subtle growth difference involving whole left side. Height 75%ile, weight 25%ile, OFC 66th %ile. MRI- hemimegancephaly and hypertrophy on one side. Mild dilation of lateral ventricles, mildly smaller left hemisphere with suggestion of pachygyria near anterior temple lobes.	*PHF6*	Stop gain SNV	NM_001015877:c.C820T:p.R274^a^	X	133549136	het	PVS1, PS2, PM2, PP3 = pathogenic	Sufficient to cause disease	COSM144567, COSM1134629
59	Between 10 and 15 years old	Moderate developmental delay, facial dysmporphisms, seizure reported at 12 years. Enlarged labia. Self-abusive when angry. Height <25%ile, weight between 50th and 75th %ile. OFC 25th %ile. CT- mild ventriculomegaly.	*SPRY4*	Nonsynonymous SNV	NM_001127496:c.T508A:p.C170S	5	141694166	het	PS2, PM2, PP3 = Likely pathogenic	Possibly contributory to brain phenotype	
			*AP4E1*	Nonsynonymous SNV	NM_001252127.1:c.T3140C:p.L1047P NM_007347.4:c.T3365C:p.L1122P	15	51294810	het	N/A	N/A	
			*AP4E1*	Splice-donor SNV	NM_001252127.1:c.121 + 2 T > C NM_007347.4:c.346 + 2 T > C	15	51207770	het	N/A	N/A	
45	Between 10 and 15 years old	Developmental delay and visual inattentiveness noted at 3 months. Athetoid movements with dystonic posturing present by 15 months and seizures noted by 2 years of age. At age four, a diagnosis of autism was suspected but could not be confirmed given the severe to profound ID. MRI: thin corpus callosum, increased ventricle and subarachnoid space size.	*CACNB3*	Nonsynonymous SNV	NM_001206915:c.G1289A:p.R430Q	12	49221639	het	PS2, PP3 = Uncertain significance	May play a role in the brain morphological phenotype	
			*SCN3A*	Nonsynonymous SNV	NM_006922.3:c.T626C:p.L209P	2	166020196	het	N/A	Selected as candidate for epilepsy phenotype. Functional studies underway	
51^b^	Between 15 and 20 years old	Significant intellectual disability. Gross motor delay. Seizuring. Scoliosis. Some hearing deficiency. Astigmatism and far-sightedness. Remarkable family history. Pregnancy complicated by possible oligohydramnios. Suctioned for meconium and physically stimulated. Placenta was calcified. MRI- asymmetrical lateral ventricles.	*SQSTM1*	Two base indel, causing a stop-gain	NM_003900: c.115_116delinsTA:p.A39^a^	5	179248051-179248052	het	PS2, PM2, PP3 = Likely pathogenic	Unsure of relative contribution of this variant versus others in the same child	
			*UPF1*	Two base indel causing a mis-sense mutation	NM_002911: c.1576_1577delinsAA:p.A526N	19	18966765 - 18966766	het	PS2, PM2, PP3 = Likely pathogenic	Unsure of relative contribution of this variant versus others in the same child	
42	Less than 5 years old.	Recurrent aspiration. Optic nerve dysfunction detected by absence of light reflex. Height, weight and OFC all at 25%ile. CT- absence of corpus callosum.	*LRP2*	Nonsynonymous SNV	NM_004525.2:c.G4351T:p.V1451F	2	170094756	het	N/A	N/A	
			*LRP2*	Nonsynonymous SNV	NM_004525.2:c.A12725G:p.D4242G	2	170003335	het	N/A	N/A	
41		CT- cerebellar atrophy									
55		CT- mild dilation of the lateral ventricles									

*Abbreviations*: *ID* Intellectual Disability, *OFC* occipito-frontal circumference, *CT* computerized tomography scan, *MRI* magnetic resonance imaging scan, *PVS1* null variant in a gene where LoF is a known mechanism of disease, *PS2* *de novo* in a patient with the disease and no family history, *PM2* absent from controls in exome sequencing project, 1000 genomes project or exome aggregation consortium, *PP3* multiple lines of computational evidence support a deleterious effect on the gene or gene product

^a^Age at examination is given in 5 year intervals in order to protect patient anonymity

^b^Patient 51 also bears a *de novo* likely contributory CNV as detailed in the text, in addition to the SNVs given here

#### *ARID1B* c.1595delG (p.G532fs) in Patient 43

This single base deletion in exon 2 of the known ID gene *ARID1B* causes a frame-shift leading to predicted loss of function (LoF, Additional file 5: Figure S1). Our patient presents with ID, autism, absence of corpus callosum, absence of speech, feeding difficulties and failure to thrive (Table 1). Haploinsufficiency of *ARID1B* was reported to cause corpus callosum abnormalities, ID, speech impairment and autism [19], suggesting the *ARID1B* LoF is causative and sufficient in this case.

#### *PHF6* c.820C > T (p.R274*) in Patient 58

*PHF6* encodes the plant homeodomain finger protein 6. The nonsense variant in *PHF6* is located in the ePHD2 domain in which causative *de novo* truncating and missense variants for Börjeson-Forssman-Lehmann syndrome (BFLS) [MIM:301900] [20], and Coffin-Siris syndrome (CSS) [MIM:135900] [21] are known. *De novo* truncating and other mutations in *PHF6* are reported to cause a distinct syndrome in girls [22] and reported for a female specific form of BFLS [23]. Roles for PHF6 are reported in the chromatin remodeling SWI/SNF complex [24], and in the NuRD epigenetic regulatory complex where it acts as a possible regulator for the latter in neurogenesis [25]. RNAi knock down of PHF6 profoundly impairs neuronal migration in vivo [26], thus leading to formation of white matter heterotopias. In keeping with this, this patient reports pachygyria, which results from abnormal migration of neurons in the developing brain. She also presents with an unusual asymmetrical growth phenotype that was reported in the one patient with the female specific BFLS [23]. These data indicate the variant is a good candidate in this case.

#### *SPRY4* c.508 T > A (p.C170S) in Patient 59

*SPRY4* encodes a specific inhibitor of the mitogen-activated protein kinase family. *Spry4* is expressed in the mouse developing brain [27], and is essential for the normal morphogenesis and cytoarchitecture of the cerebellum [28]. Morphogenic changes in axon growth have been shown when the protein is down regulated both in vivo and in vitro [29]. In zebrafish, *spry4* is a principal regulator of mid-brain development [30], and mediates hindbrain patterning [31]. These data support the notion that the *SPRY4* missense variant may contribute to the brain morphological phenotype in this patient. *Spry4* expression plays a role in Xenopus limb bud development [32], of note as our patient reports short and crowded toes.

#### *CACNB3* c.1289G > A (p.R430Q) in Patient 45

*CACNB3* encodes a regulatory subunit of a voltage-dependent calcium channel (VDCC). Mice lacking *Cacnb3* presented visual impairment [33], high pain threshold [34], and behavioral phenotypes [34], all of which features are seen in this patient. Mutations in other members of VDCC subunit encoding genes are known to cause neurological disease, including epilepsy [35] present in our patient. This variant is found in eight of 60,165 individuals in the ExAC database, where its non-absence disqualifies likely pathogenicity as per ACMG criteria, despite being *de novo* and deleterious by multiple lines of computational evidence. Neither does it meet criteria to be a benign variant, and therefore is of uncertain significance.

#### *SQSTM1*c.115_116delinsTA (p.A39*), *UPF1* c.1576_1577delinsAA *(p.A526N)* and a 1q43(1:243282457–243447771, hg19) deletion CNV in Patient 51

The patient is severely affected, with significant ID and several major congenital anomalies (Table 1). The heterozygous indel formed by two adjacent SNVs in *SQSTM1* causes a stop-gain. *SQSTM1* encodes p62, a regulatory factor in Nuclear Factor kappa-B (NF-kB) signaling, NF-E2-related factor 2 (NRF2) activation, ubiquitin-mediated authophagy, and transcription [36]. The SNV is located in the PB1domain, mutations of which cause Paget Disease of Bone (PDB) and Frontotemporal Dementia and/or Amyotrophic Lateral Sclerosis (FTLD/ALS) [MIM:607485,612069] [36]; both neurodegenerative conditions that include morphological brain changes. The adjacent SNVs in *UPF1,* together cause a likely pathogenic missense amino acid change (Table 1 and Additional file 5: Figure S1). UPF1 has an essential role in nonsense-mediated mRNA decay [37]. Interestingly, UPF1 has been shown to remarkably reduce ALS-associated neuronal toxicity in vitro [38] and to protect against motor dysfunction and forelimb paralysis in a rat model for ALS [39]. It is plausible haploinsufficiency of *SQSTM1* may have caused neurofunctional defects, which the haploinsufficiency of *UPF1* may have exacerbated. In this regard, it is notable that at 19 years of age, patient 51 presents significant motor deficits, being wheelchair bound, indicative of a possible early onset of ALS. While scoliosis and hearing loss, both among the presentation for PDS is already seen in her. These data support the notion that the SNVs in both genes maybe contributory toward her presentation.

**Fig. 2:**
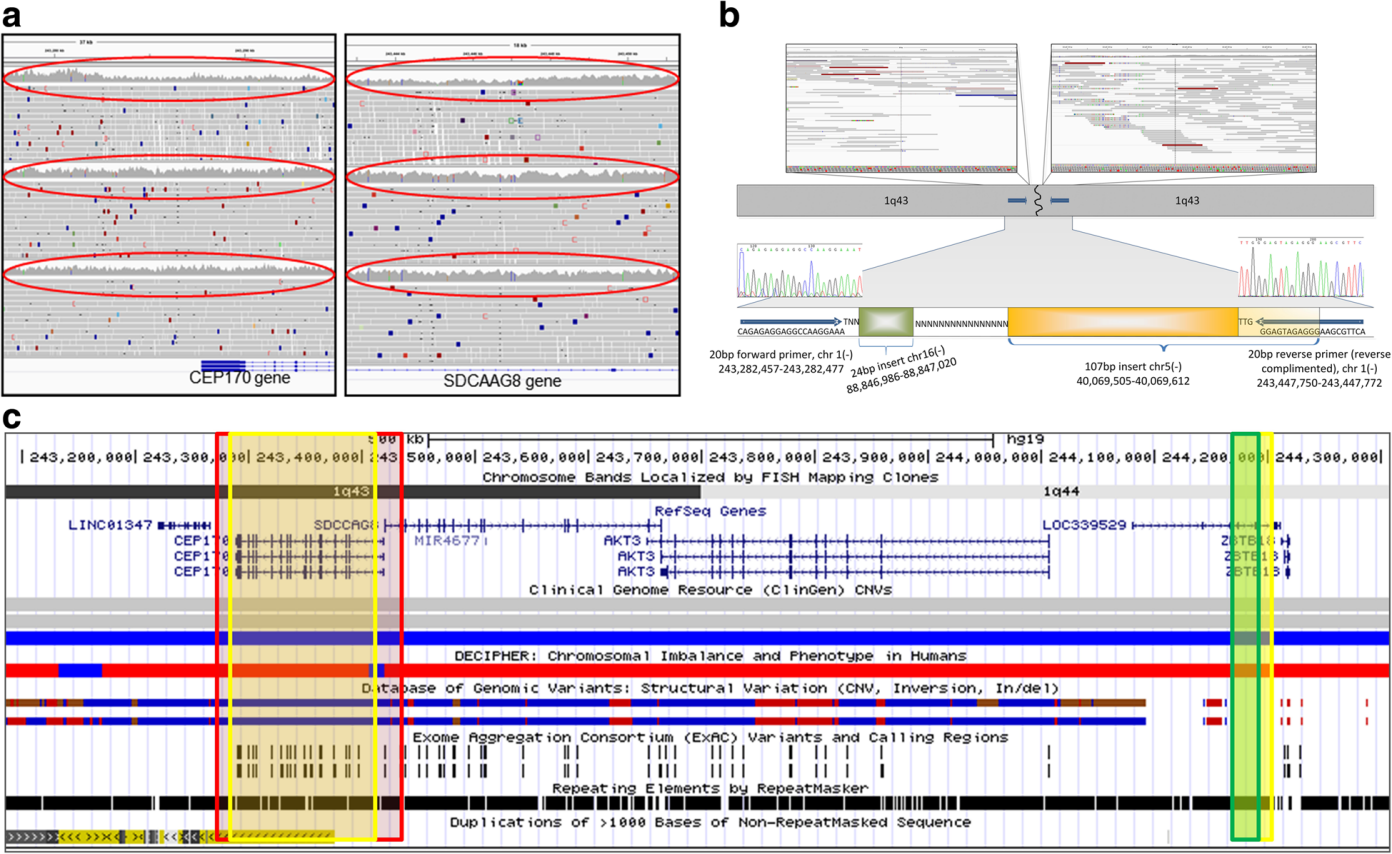
Details of CNV analyses. **a** IGV images for heterozygous deletion CNV in patient 51, showing proximal and distal breakpoint. The CNV involves whole of *CEP170* and part of *SDCAAG8* genes. Top, middle and bottom panels are child’s .bam file, mother’s .bam file and father .bam file respectively. Read-depth coverage shows CNV is *de novo* (*red ovals*). **b** Cartoon of breakpoint junction seuqence showing a 24 bp chromosome 16 (*green box*) and 107 bp chromosome 5 sequence (*yellow box*) inserted between the proximal and distal breakpoints on chromosome 1q43. Yellow shaded segment shows sequnce microhomology- this 14 bp seuqence (TTGGGAGTAGAGGG) is found at chromosome 5:40,069,598-40,069,612 and at chromosome 1:243,447,747-243,447,761, hg19). Sanger sequence trace images are overlaid confirming the CNV breakpoint. *Grey arrows* denote PCR forward and reverse primers. N denotes DNA repeat sequence. **c** Genomic interval involved in the *de novo* CNV detected in patient 51- ucsc genome browser (hg19). *Red highlighted box* shows region involved in the deletion in our patient. *Yellow boxes* show critical region for 1q43-44 sydrome defined by Nagamani et al. *Green box* shows critical region as defined by Perlman et al. N.B, Nagamani et al. also highlight *ZBTB18* (old name *ZNG238*) in their critical region

We further verified a *de novo* ~165 kb heterozygous deletion that spans *CEP170* [MIM:613023] in whole and *SDCCAG8* [MIM:613524] in part (Fig. 2a and c) in this patient. *CEP170* encodes a component of the centrosome [40]. SDCCAG8 is also involved in centrosome function [41], DNA damage response signaling [42] and neuronal migration [41]. Both genes are suggested as candidates for corpus callosum abnormalities via 1q43 microdeletion [43], however this has been contested [44] (Fig. 2c). Our patient presents partial phenotypic overlap with microdeletion 1q34 index cases. The demonstrated roles for SDCCAG8 in DNA-mismatch repair, and for both genes in cell cycle progression, supports the notion this CNV may be contributory. Notably, the haploinsufficiency of a DNA-mismatch repair gene could lead to the high mutation burden detected in this child (above SNVs, and *vide* section ‘Genome Assembly Indels’). We also confirmed at least one maternally inherited balanced translocation (*vide* section on CNV/SVs), which is unlikely to be contributory.

### Mutation burden assessment in large secondary positive and negative control cohorts support candidacy of novel genes

We investigated the candidacy of the above verified genes by assessment for incidence of damaging mutation in large positive and negative cohorts with comparable NGS data. We looked for ‘potentially damaging SNVs’ (PDSs) (Additional file 5: Figure S2 gives an example per patient PDS mutation burden) in our candidate genes, from WES of 2081 patients with neurodevelopmental and neurocognitive phenotypes from the UK10K cohort [15] (Additional file 4: Table S3 and Additional file 5: Figure S3) and compared that to incidence in WGS from 2535 healthy people from the 1000 Genomes project [16].

**Fig. 3:**
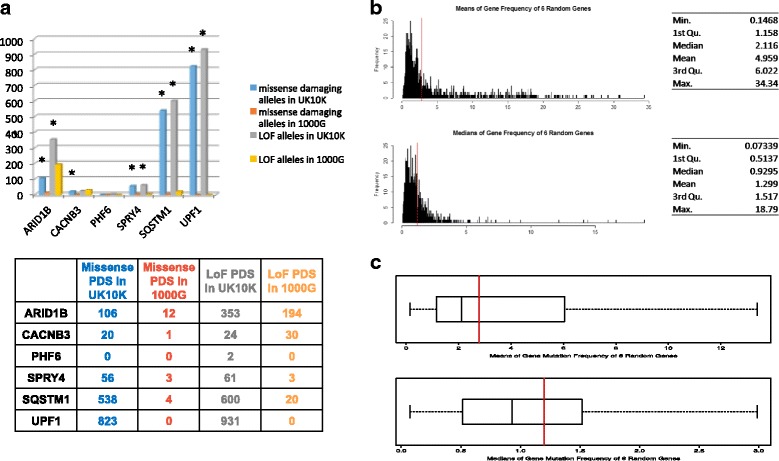
Validation study. **a** showing incidence for potentially damaging SNVs (PDSs) in both the positive control (UK10K) and negative control (1000G) control cohorts. * denotes statistical signficance (at *p* < 0.05, Fisher’s exact test) **b** and **c** Results of bootstrap analyses for PDSs in 6 randomly selected genes. *Red vertical bar* shows the mean and median result for PDSs in our 6 candidate genes

We first screened for the exact variant detected in our discovery cohort, and did not find any case of an exact match. We then conducted a gene-wise PDS screen and observed that incidence for PDS in *ARID1B, SPRY4, CACNB3, SQSTM1* and *UPF1* were significantly enriched in the positive versus negative control cohorts (Fig. 3a). There was no significance for *PHF6*; however, the two PDS found in 4616 people was insufficient for meaningful statistical assessment. The extremely high PDS burden in the positive control cohort for *SQSTM1* and *UPF1* is noteworthy, as these genes have previously not been reported for ID to our knowledge, and further, the indels in both are found in the same patient in our cohort.

While we do not have access to clinical data to conduct a classical genotype-phenotype correlation between cases in the positive control cohort and our patients who have the same gene affected, the large number of such cases in the positive control cohort also impedes such a study within the scope of this work. We therefore assessed if our findings could be due to random chance effect, by bootstrapping the UK10K cohort for PDS in six randomly selected genes each, a thousand times. We found from the bootstrap analysis that the mean and median gene-wise variant frequency for our six candidate genes was greater than that of the corresponding distribution, indicating that our findings were not likely due to chance (Fig. 3b & c). These data are consistent with an association of at least five of our candidate genes with neurodevelopmental abnormalities.

### Novel candidate genes converge unto the ubiquitin proteasome pathway, which also bears significant mutation burden in 2081 positive control WES samples

We investigated molecular links between our pathogenic and candidate genes; focused IPA and STRING pathway analyses revealed that all six connected to the ubiquitin proteasome degradation pathway (UPP) (Additional file 5: Figure S4) which has important roles in the structural development and function of the brain [45, 46]. We assessed the relative importance of this pathway and found the UPP was among significantly enriched pathways for PDS when compared with all KEGG pathway categories (*n* = 55) (Additional file 7: Table S5), in the UK10K patient cohort (*p* = 0.031), substantiating the importance of the UPP pathway in brain development.

### Mendelian inheritance and N of 1 analyses provides additional candidate variants

In addition to our *in-silico* refinement and test for candidate *de novo* SNVs above, we also conducted a classical series of N of 1 studies for these eight patients; manually assessing the possible candidacy of variants selected by all possible Mendelian inheritance patterns (Additional file 8: Table S6). Compound heterozygous missense mutations were identified in *LRP2* [MIM:600073], causative of the autosomal recessive Donnai Barrow syndrome [MIM:222448] in patient 42. Absence of the corpus callosum, reported in our patient, presents in Donnai Barrow syndrome. Compound heterozygous mutations were identified in *AP4E1* [MIM:607244] causative of autosomal recessive spastic paraplegia type 51, in patient 59. This patient reported a seizure at 12 years of age, however does not exhibit the severe neurological phenotypes nor the shy demeanour reported for a possible syndromic form of ID [47, 48] caused by defects in adaptor protein complex-4, of which *AP4E1* encodes one of the four subunits. A missense *SCN3A* [MIM:182391] SNV (p.Leu209Pro/c.626 T > C) in patient 45 was selected due to SCN3A association to epilepsy [49] (a phenotype presented by our patient), and the predicted deleterious effect of the variant, and was Sanger verified as *de novo*. Functional studies are underway to further investigate the role of *SCN3A* in epilepsy.

### Extensive copy number variant (CNV) and structural variant (SV) analyses identifies likely causative CNV missed by clinical CMA, and balanced benign translocation

**Fig. 4:**
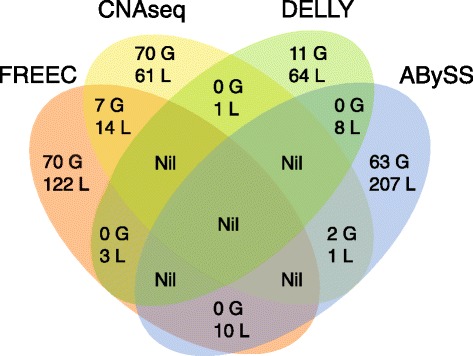
Venn driagram showing CNVs found by each algorithm (G = gain, L = loss)

We conducted both alignment-based (FREEC, CNAseq, DELLY) and *de novo* assembly-based (ABySS) CNV/SV analyses. CNVs, i.e., duplications (gains) and deletions (losses) were identified by all four platforms, while SVs, i.e. translocations and inversions, were identified by DELLY and ABySS (Table 2). An average of 58 *de novo* gain CNVs and 128 *de novo* loss CNVs across all eight patients were detected. However, only 46 CNVs were called by over one platform, and none were called by more than two (Fig. 4), with the majority of each algorithm’s findings being unique. We carried out extensive visual *in silico* curation for all CNVs, and selected three to verify of which, only the previously discussed 1q43 loss CNV, Sanger verified as *de novo*- it was detected by FREEC and CNAseq, and is clearly visible on IGV (Fig. 2a). Breakpoint junction sequence reveals a complex architecture (Fig. 2b).

**Table 2:** Number of copy number variants and structural variants identified

Patient #	FREEC		CNAseq		DELLY				ABySS			
	Gains	Losses	Gains	Losses	Gains	Losses	Inv	Trans	Gains	Losses	Inv	Trans
42	10	29	8	11	0	0	0	1	4	11	1	8
55	8	22	17	7	0	0	0	2	0	5	1	2
58	8	18	5	8	0	2	0	1	2	10	2	1
41	14	13	13	16	10	60	16	19	4	23	0	4
59	8	18	7	10	0	2	0	2	4	17	2	11
43	14	18	5	5	0	3	0	0	16	25	1	5
51	6	19	13	11	0	0	0	0	30	113	5	21
45	9	12	11	9	1	1	0	1	5	14	2	7
Totals	77	149	79	77	11	68	16	26	65	218	14	59

Similar to our CNV results, SV results from DELLY and ABySS were divergent (Table 2). Only one translocation (between chromosome 19 and 1) in patient 41, was called by both, and there was no concordance among inversions. Upon extensive manual *in silico* curation we selected 10 translocations and 1 inversion to verify (Additional file 9: Table S7), but none verified as *de novo*. Sanger verification for these lesions was challenging as breakpoints mapped to repeat-masked regions, nevertheless one translocation verified as maternally inherited; a chromosome X-2 (92696685:225020555, hg19) translocation not causing any gene-disruption, in patient 51. The breakpoint junction shows a single base addition (Fig. 6a).

### Genome assembly yields small insertions/deletions (indels) missed by genome re-alignment

In contrast to the re-alignment based algorithms, ABySS [13] identified over 700 potential *de novo* indels (maximum size 100 bp), via genome assembly. Forty three indels were refined as likely true positives with a functional importance, due to having at least seven spanning reads, and producing a protein coding change; the majority being in patient 51. For consistency, we conducted a pathway analyses for the indel-bearing genes, and a manual curation, in order to select candidates for verification as we had done for our SNVs. This resulted in 14 indels that were Sanger tested (Additional file 1); however one was false positive, five were inherited, and eight did not pass PCR quality checks (Additional file 9: Table S7), indicating location to repeated DNA sequence, thus hampering any ability to amplify the region for Sanger sequencing.

### Gene regulatory region variation identified in known and hypothesized ID genes

We investigated gene regulatory sequence variation which we term ‘*de novo* variants in possible regulatory regions’ (DVPRRs). We filtered the DVPRR for potentially pathogenic changes using two approaches: by screening for involvement in known ID genes, and on the basis of our hypothesized involvement of the UPP.

**Fig. 5:**
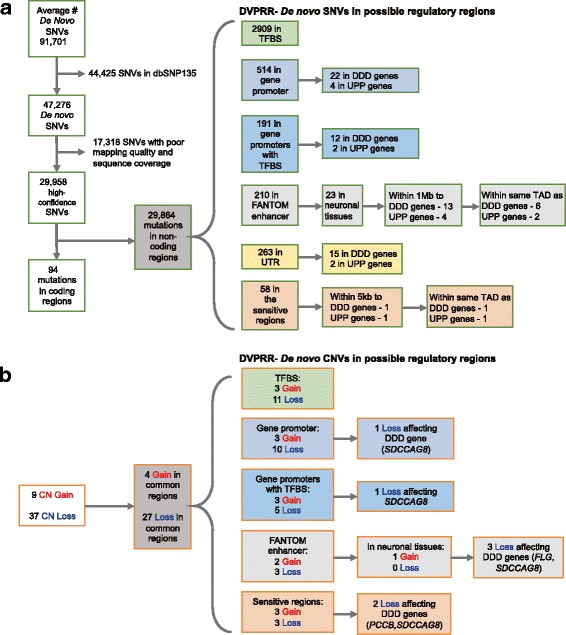
Schematic of filtration pipeline for variants in non-coding regions. **a** Schematic for SNVs. **b** Schematic for CNVs. Abbeviations; SNV- single nucleotide variant, TFBS – Transcription Factor Binding Site, FANTOM-Enhancer sequence as annotated by the Fantom consortium. UTR – untranslated regions. DDD- Deciphering Development Disabilities. UPP – Ubiquitin proteosome degredation pathway. CN- copy number. Patient 42 had DVPRR in the UTRs of two genes; *CBL* and *UBE3B*. Patient 59 had a DVPRR in the promoter of *UBE3A*, patient 43 had a DVPRR in the promoter of *CUL4B*, and patient 42 had DVPRRs in the promoters of *UBE3A, CUL4B* and *CUL7* (Additional file 10: Table S8)

An average ~30,000 *de novo* SNVs were found across our eight patients in the non-coding genome (Fig. 5a). Of these, an average 2909 located to transcription factor binding sites, an average 514 to putative gene promoters, an average 191 of those located to promoters were also located to transcription factor binding site regions, an average 210 located to regions annotated as enhancers by the FANTOM consortium [50], an average 263 belonged to 5′ or 3′ UTR regions and an average 58 located to highly conserved ultra-sensitive regions [51] – we considered these to be DVPRR and therefore there were an average 3763 DVPRR across all eight patients (Fig. 5a). We then intersected DVPRRs with 995 genes known to cause developmental delay (‘DDD genes’) [52] in a disease gene screen approach, and with the total 137 genes of the UPP (KEGG), − as our candidate genes converged upon the UPP - in a hypothesis-driven approach. As a final step for enhancers and ultra-sensitive regions, we further selected DVPRR where it, and the candidate gene (DDD genes or UPP genes), were located within the same topological domain [53], postulating that their physical proximity would imply that the regulatory region in question did in fact impact the targeted gene. In summary we found an average of 56 and 11 DVPRR per patient in our gene-screen and hypothesis driven approach respectively, by these filtrations combined (Additional file 10: Table S8) (Fig. 5a). We also interrogated high-confidence CNVs in the same manner, but only found association to *SDCCAG8*, a known ID gene present in the previously discussed 1q43 microdeletion (Fig. 5b and Additional file 10: Table S8).

### Occurrence of *de novo* SNVs in non-coding RNAs (ncRNA)

We found an average of 241 high confidence *de novo* SNVs that located to sequence annotated as ncRNA across all eight patients. A majority of these (average 195) fall within introns while an average 39 are exonic, an average 0.25 are predicted in splice junction sequence and average 5 and 2 are located to 3′ and 5′ UTR respectively.

## Discussion

### Selection of candidate SNVs: comparison of strategies

An effective strategy is essential to select causative SNVs from NGS data. Standard filtration approaches (e.g., variant quality, mapping quality, minimum read depth, and functional variants that are not common polymorphisms) yield potential *de novo* variants that then must be careful sifted for likely true candidates. In keeping with others [54], we found an average of 6 +/− 2 candidate unverified *de novo* SNVs (Additional file 8: Table S6), and it was necessary to implement an effective prioritization approach for verification.

Discovery WGS and WES studies published to date have used a large sample size [2], detailed pedigree information [55], or well characterized rare syndromes [56] as study cohorts, leveraging the power of numbers, inheritance pattern, and phenotypic commonality, respectively, as filtration strategies. In as much as we did not have a large cohort, all of our cases were sporadic, and none had a recognized dysmorphic syndrome, we refined SNVs objectively, by selecting genes known to be involved in brain development pathways. We reasoned that this systematic approach would reduce subjective bias inherent in an N of 1 genotype-phenotype correlation, and thereby identified potential candidates. However a subjective screen for SNVs yielded the likely damaging variant in *SCN3A*, which was not stratified by our objective approach –highlighting the limitation of pathway analyses programs that depend on available gene-functional annotations. Notably *SCN3A* was selected by a team of biochemical geneticists specifically with respect to the epilepsy presented by the child. Thus a subjective approach may also miss results detected from objective screening, as exemplified in this case, where the two analyses were done by independent members and each did not report the result of the other.

### Interpreting detected variants; discovery study findings further inform genetic complexity for ID

Variable expressivity and reduced penetrance are well known in the pathogenicity of ID, and it is increasingly recognized that a single mutation in a single gene may only rarely explain the full phenotypic spectrum [1]. Our results provide further indications of such complex heritability; in patient 51, the 1q43 deletion, and SNVs in *SQSTM1* and *UPF1* may act in concert to produce the complex and severe phenotype in this patient. While in patient 58, we have identified both compound heterozygous variants in the known ID gene *AP4E1* that act in a recessive model, as well as *de novo* variant in a novel gene *SPRY4*, which has important functions in brain development. *De novo* mutation is recognized to play an important role particularly in the pathogenicity of ID [57], and it is difficult to determine to what extent each of these variants, if at all, contributes to disease burden in this patient. The same is true for patient 45 in whom *de novo* variants for two novel genes, *CACNB3* and *SCN3A* were identified. We note that patient 51 who bears the most complex genotype, is the most severely affected in our cohort, and in this case, clinical severity does co-relate with number and complexity of genomic alterations, suggesting that gradation of clinical severity may provide useful toward assessing the contribution of genomic alterations.

It is recognized that genes responsible for ID converge onto common networks [1, 58]. The candidate genes we identified converge onto the UPP, which is critically involved in neurodegenerative disease [45] and has important roles in neurodevelopmental disorders [45, 46]. This observation is consistent with the notion that they may be good candidates, and exemplifies the usefulness of probing molecular links among novel findings.

### Large secondary positive WES cohort analysis supports novel findings

Novel SNV findings from NGS studies require rigorous additional studies to support proof of pathogenicity [14]. In our case, several of our novel candidates cause missense variation, whose effect is difficult to model, as opposed to clear loss of function mutations which are amenable to functional studies in model organisms. Conversely we were unable to conduct traditional genotype-phenotype correlations studies as none of our patients had a recognizable syndrome to match with other patients. Therefore, our approach of using a large secondary positive control cohort, despite the phenotypic spectrums not matching our cases precisely, gave us sufficient ability to test the predicted causality of our candidate genes and was the best strategy available. We were hampered by the lack of an optimal comparison negative control cohort. We used WGS data from the 1000 genomes project, which we recognize is primarily comprised of low coverage samples whose phenotypic spectrum is poorly characterized (thus yielding likely false negative data or conversely identifying variants in ‘normal’ individuals who are in fact affected), yet the similarity of sample size between the two groups allowed us to explore the PDS distribution for these genes reasonably, providing a useful contributory analysis toward assessing their likely pathogenicity. Finally this large cohort enabled us to further probe the convergence of our candidate genes upon UPP, by assessing its contribution versus other biological pathways.

### WGS is able to detect structural variants below the threshold of clinical CMA, and enables mechanistic insights into CNV formation

By using WGS instead of WES, we were able to detect a CNV below clinical CMA resolution, isolate it’s breakpoints, and uncover a possible complex genomic landscape in one patient. We wanted to conduct a comprehensive screen for CNVs and other structural variants to maximize sensitivity. Therefore we used four approaches that are fundamentally different; CNAseq and FREEC are sequence based copy-number estimators that use categorically different algorithmic approaches for background correction. DELLY is an alignment based assembler, whilst ABySS is a *de-novo* genome assembler. Since each algorithm was optimized differently, it therefore yielded different results. For example, CNAseq executes read-depth based binning, and hence aggregates results at telomeres and centromeres where a larger number of reads re-align due to pervasive repeat sequence (Additional file 5: Figure S5). The verified CNV we detected was only identified by DELLY and FREEC, but missed by the other algorithms. Therefore, we caution against using only one CNV detection algorithm as this would reduce sensitivity. The breakpoint junction sequence in the case of the confirmed 1q43 microdeletion is consistent with the notion that it could be caused by chromotripsis, a mechanism only recently reported in the constitutional genome [59], further demonstrating the utility of WGS data.

### WGS enabled a *de novo* genome assembly that unmasked hidden genome complexity

**Fig. 6:**
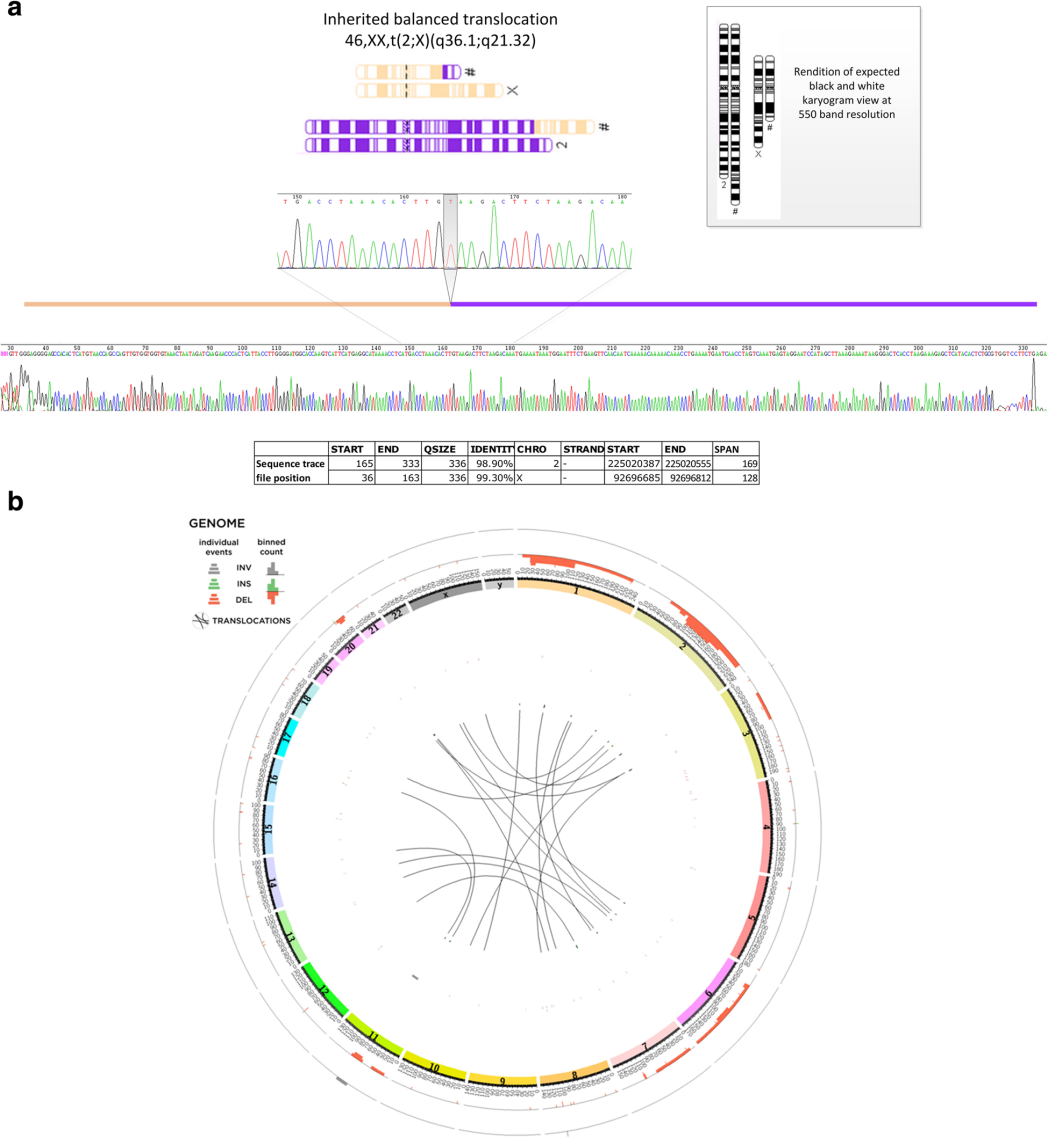
**a** Sanger sequencing verification of translocation in patient 51, with karyotype cartoon of balanced translocation. PCR amplicon trace file shows sequence mapping across the chromosome X -2 translocation boundary. Zoomed-in view shows single base addition at breakpoint jucntion. **b** Circos plot showing mutation burden for patient 51 called by ABySS genome assembly

ABySS *de novo* assembly identified a translocation missed by DELLY, and also detected a higher than usual number of putative indels in patient 51, who was found to have a remarkably unstable genome masked by standard genome re-alignment based analysis (Fig. 6b). However, we experienced difficulty confirming these events via Sanger sequencing, which was due, in part, to the high degree of repeated sequence at breakpoint junctions. Genome assembly is able to call events in repetitive sequence better than alignment based algorithms [13], though conversely such events are harder to independently verify. We are among the first to use *de novo* assembly to interrogate patients with ID, and our findings suggest variation located to repeat enriched sequence is currently under-ascertained in the constitutional genome.

### WGS is able to interrogate regulatory genomic sequence

Meaningful interpretation of SNVs within regulatory sequence is hampered by the sparsity of annotations for the non-coding genome. We implemented two different filtering strategies in order to identify non-coding SNVs that could have a functional impact, and also used topological domain data to further refine good candidates. Though we were able to reduce the number of candidate DVPRR from an average >3700 to dozens in the case of our gene-screen approach and a handful in the case of our hypothesis driven approach, nonetheless without further focused studies, meaningful interpretations are precluded. In contrast, assessing the impact of CNV-based DVPRR is theoretically less challenging, as it is more straightforward to predict functional outcome for a complete loss or gain of a possible regulatory sequence. In summary, though clinically relevant conclusions for DVPRR will require a case-by-case analysis and extensive follow-up functional studies, nevertheless we note it is possible to stratify DVPRR in the context of known causative genes for ID using WGS.

### WGS versus WES

WGS yields a comprehensive screen of the genome as, in addition to coding variation, it includes ability to investigate structural variation at a fine scale as discussed above, and also variation in possible gene regulatory sequence as well as ‘non-coding genes’ such as ncRNAs for which there is a paucity of information in the context of neurodevelopmental disease. While we show strategic stratification for DVPRR can yield results potentially relevant to ID causation, much less is possible for annotation of SNVs within ncRNA sequence, of which we detect an average 241 across our samples. Nevertheless, initial screens such as ours, importantly generate exploratory information for non-coding sequence variation possible only by WGS.

We note that all the SNVs we identified as involved in disease would have been possible to detect by WES. However, WGS yields a more complete view of possible pathogenic variation in each child. This is exemplified in the case of patient 51, for whom had only WES been performed, while the *SQSTM1* and *UPF1* SNVs would likely have been detected, the 1q43 microdeletion would not have been identified. In the case of this patient, it is unclear what the gene-effect size for each variant is. Conversely, in the case of patient 43, for whom we detected the SNV in *ARID1B*, we are more certain of the penetrance of this variant due to the normal results for other causative variation in their genome (i.e., that they do not have any CNVs or SVs) from our WGS data analyses. These data argue in favor of WGS over WES for clinical use.

## Conclusions

This is the first study to present extensive analyses of WGS data in the context of ID, for causative SNV and CNV/SV in both coding and non-coding sequence, and the first to present results from *de novo* assembly of the genome. In a heterogeneous group of eight children with ID and morphological brain defects, we were able to identify candidate causative variants, highlight neurodevelopmental pathways, and unearth hidden genome instability, demonstrating the efficacy of a discovery approach to WGS analyses in the context of ID.

## Additional files

Additional file 1: Supplementary Methods - Additional details on methods presented succinctly in main text. (DOCX 53 kb)

Additional file 2: Table S1. Test of relatedness - Table showing relatedness for each trio by comparing SNP concordance between child, mother and father. (XLSX 17 kb)

Additional file 3: Table S2. Primers for verification - All primer sequences used for SNV, CNV and SV verification. (XLSX 18 kb)

Additional file 4: Table S3. UK10K study cohorts that comprise the positive control cohort – Table giving descriptions of the study cohorts from the UK10K project that comprised the positive control cohorts and conditions for use. (XLSX 13 kb)

Additional file 5: Supplementary Figures. **Figure S1.** IGV image and Sanger verification trace files for indel in *ARID1B* and missense variation in *UPF1*. **Figure S2.** UK10K mutation load – counts as per variant annotation type on one patient. **Figure S3.** Histogram of mutation burden per patient in the UK10K cohort. **Figure S4.** Pathway interactions showing convergence onto UPP pathway. **Figure S5.** Plots for CNV distribution for two chromosomes as called by CNAseq. (DOCX 1972 kb)

Additional file 6: Table S4. Detailed variant information for all verified variants, including ACMG classification – Table showing details for variant classification such as pathogenicity prediction algorithms results and details of ACMG criteria application. (XLSX 18 kb)

Additional file 7: Table S5. Significantly enriched KEGG pathways for PDS burden in positive control cohort – Details for pathway enrichment analysis showing all KEGG pathways and burden of enrichment for each in the positive control cohort. (XLSX 15 kb)

Additional file 8: Table S6. Raw data for SNVs detected across all patients by Mendelian Inheritance Pattern filtering – all SNVs detected per trio, organized as one trio per sheet, using the Mendelian inheritance filtering. Sheet 1 gives the legend. All SNVs are annotated, including CADD and RVIS scores. (XLSX 57 kb)

Additional file 9: Table S7 CNV/SV/Indel Verification Summary – Table giving a list of all indels, CNVs and SVs (inversions and translocations), and details on their verification. (XLSX 15 kb)

Additional file 10: Table S8. Summary of SNVs and CNVs across 8 ID patients in DVPRR – Details on all SNVS and CNVs detected in DVPRR for all patients, organized by those intersecting with DDD genes and those with UPP genes. (XLSX 40 kb)

## Abbreviations

1000G project: http://www.internationalgenome.org/
CMA: Clinical microarray
CNV: Copy number variant
dbSNP135: https://www.ncbi.nlm.nih.gov/snp
DGV: Database of Genomic Variants
DGV: http://dgv.tcag.ca/dgv/app/home
DVPRR: de novo variants in possible regulatory regions
FANTOM consortium: http://fantom.gsc.riken.jp/
GRCh37-lite/hg19a: http://www.bcgsc.ca/downloads/genomes/9606/hg19/1000genomes/bwa_ind/genome/README.GRCh37-lite
KEGG: www.genome.jp/kegg/pathway.html
NGS: Next generation sequencing
NHLBI-ESP: http://evs.gs.washington.edu/EVS/
OMIM: www.omim.org
PDS: Potentially damaging SNVs
Picard: https://broadinstitute.github.io/picard/
SNV: Single nucleotide variant
STRING: www.string-db.org
SV: Structural variant
UK10K project: www.uk10k.org
UPP: Ubiquitin proteasome pathway
WES: Whole exome sequencing
WGS: Whole genome sequencing

## References

[1] Vissers LE, Gilissen C, Veltman JA (2016). Genetic studies in intellectual disability and related disorders. Nat Rev Genet.

[2] Gilissen C, Hehir-Kwa JY, Thung DT, van de Vorst M, van Bon BW, Willemsen MH, Kwint M, Janssen IM, Hoischen A, Schenck A (2014). Genome sequencing identifies major causes of severe intellectual disability. Nature.

[3] Danecek P, Auton A, Abecasis G, Albers CA, Banks E, DePristo MA, Handsaker RE, Lunter G, Marth GT, Sherry ST (2011). The variant call format and VCFtools. Bioinformatics.

[4] Fejes AP, Khodabakhshi AH, Birol I, Jones SJ (2011). Human variation database: an open-source database template for genomic discovery. Bioinformatics.

[5] Sim NL, Kumar P, Hu J, Henikoff S, Schneider G, Ng PC (2012). SIFT web server: predicting effects of amino acid substitutions on proteins. Nucleic Acids Res.

[6] Pollard KS, Hubisz MJ, Rosenbloom KR, Siepel A (2010). Detection of nonneutral substitution rates on mammalian phylogenies. Genome Res.

[7] Adzhubei IA, Schmidt S, Peshkin L, Ramensky VE, Gerasimova A, Bork P, Kondrashov AS, Sunyaev SR (2010). A method and server for predicting damaging missense mutations. Nat Methods.

[8] Schwarz JM, Rodelsperger C, Schuelke M, Seelow D (2010). MutationTaster evaluates disease-causing potential of sequence alterations. Nat Methods.

[9] Kircher M, Witten DM, Jain P, O’Roak BJ, Cooper GM, Shendure J (2014). A general framework for estimating the relative pathogenicity of human genetic variants. Nat Genet.

[10] Boeva V, Popova T, Bleakley K, Chiche P, Cappo J, Schleiermacher G, Janoueix-Lerosey I, Delattre O, Barillot E (2012). Control-FREEC: a tool for assessing copy number and allelic content using next-generation sequencing data. Bioinformatics.

[11] Jones SJ, Laskin J, Li YY, Griffith OL, An J, Bilenky M, Butterfield YS, Cezard T, Chuah E, Corbett R (2010). Evolution of an adenocarcinoma in response to selection by targeted kinase inhibitors. Genome Biol.

[12] Rausch T, Zichner T, Schlattl A, Stutz AM, Benes V, Korbel JO (2012). DELLY: structural variant discovery by integrated paired-end and split-read analysis. Bioinformatics.

[13] Simpson JT, Wong K, Jackman SD, Schein JE, Jones SJ, Birol I (2009). ABySS: a parallel assembler for short read sequence data. Genome Res.

[14] Richards S, Aziz N, Bale S, Bick D, Das S, Gastier-Foster J, Grody WW, Hegde M, Lyon E, Spector E (2015). Standards and guidelines for the interpretation of sequence variants: a joint consensus recommendation of the American College of Medical Genetics and Genomics and the Association for Molecular Pathology. Genet Med.

[15] Consortium UK, Walter K, Min JL, Huang J, Crooks L, Memari Y, McCarthy S, Perry JR, Xu C, Futema M (2015). The UK10K project identifies rare variants in health and disease. Nature.

[16] Genomes Project C, Auton A, Brooks LD, Durbin RM, Garrison EP, Kang HM, Korbel JO, Marchini JL, McCarthy S, McVean GA (2015). A global reference for human genetic variation. Nature.

[17] Kanehisa M, Goto S (2000). KEGG: kyoto encyclopedia of genes and genomes. Nucleic Acids Res.

[18] Tucker T, Zahir FR, Griffith M, Delaney A, Chai D, Tsang E, Lemyre E, Dobrzeniecka S, Marra M, Eydoux P (2014). Single exon-resolution targeted chromosomal microarray analysis of known and candidate intellectual disability genes. Eur J Hum Genet.

[19] Halgren C, Kjaergaard S, Bak M, Hansen C, El-Schich Z, Anderson CM, Henriksen KF, Hjalgrim H, Kirchhoff M, Bijlsma EK (2012). Corpus callosum abnormalities, intellectual disability, speech impairment, and autism in patients with haploinsufficiency of ARID1B. Clin Genet.

[20] Lower KM, Turner G, Kerr BA, Mathews KD, Shaw MA, Gedeon AK, Schelley S, Hoyme HE, White SM, Delatycki MB (2002). Mutations in PHF6 are associated with Borjeson-Forssman-Lehmann syndrome. Nat Genet.

[21] Wieczorek D, Bogershausen N, Beleggia F, Steiner-Haldenstatt S, Pohl E, Li Y, Milz E, Martin M, Thiele H, Altmuller J (2013). A comprehensive molecular study on Coffin-Siris and Nicolaides-Baraitser syndromes identifies a broad molecular and clinical spectrum converging on altered chromatin remodeling. Hum Mol Genet.

[22] Zweier C, Kraus C, Brueton L, Cole T, Degenhardt F, Engels H, Gillessen-Kaesbach G, Graul-Neumann L, Horn D, Hoyer J (2013). A new face of Borjeson-Forssman-Lehmann syndrome? De novo mutations in PHF6 in seven females with a distinct phenotype. J Med Genet.

[23] Berland S, Alme K, Brendehaug A, Houge G, Hovland R (2011). PHF6 deletions may cause Borjeson-Forssman-Lehmann syndrome in females. Mol Syndromol.

[24] Kosho T, Miyake N, Carey JC (2014). Coffin-Siris syndrome and related disorders involving components of the BAF (mSWI/SNF) complex: historical review and recent advances using next generation sequencing. Am J Med Genet C Semin Med Genet.

[25] Todd MA, Picketts DJ (2012). PHF6 interacts with the nucleosome remodeling and deacetylation (NuRD) complex. J Proteome Res.

[26] Zhang C, Mejia LA, Huang J, Valnegri P, Bennett EJ, Anckar J, Jahani-Asl A, Gallardo G, Ikeuchi Y, Yamada T (2013). The X-linked intellectual disability protein PHF6 associates with the PAF1 complex and regulates neuronal migration in the mammalian brain. Neuron.

[27] Zhang S, Lin Y, Itaranta P, Yagi A, Vainio S (2001). Expression of Sprouty genes 1, 2 and 4 during mouse organogenesis. Mech Dev.

[28] Yu T, Yaguchi Y, Echevarria D, Martinez S, Basson MA (2011). Sprouty genes prevent excessive FGF signalling in multiple cell types throughout development of the cerebellum. Development.

[29] Hausott B, Vallant N, Schlick B, Auer M, Nimmervoll B, Obermair GJ, Schwarzer C, Dai F, Brand-Saberi B, Klimaschewski L (2012). Sprouty2 and −4 regulate axon outgrowth by hippocampal neurons. Hippocampus.

[30] Dyer C, Blanc E, Hanisch A, Roehl H, Otto GW, Yu T, Basson MA, Knight R (2014). A bi-modal function of Wnt signalling directs an FGF activity gradient to spatially regulate neuronal differentiation in the midbrain. Development.

[31] Labalette C, Bouchoucha YX, Wassef MA, Gongal PA, Le Men J, Becker T, Gilardi-Hebenstreit P, Charnay P (2011). Hindbrain patterning requires fine-tuning of early krox20 transcription by Sprouty 4. Development.

[32] Wang YH, Beck CW (2014). Distal expression of sprouty (spry) genes during Xenopus laevis limb development and regeneration. Gene Expr Patterns.

[33] Cork RJ, Namkung Y, Shin HS, Mize RR (2001). Development of the visual pathway is disrupted in mice with a targeted disruption of the calcium channel beta(3)-subunit gene. J Comp Neurol.

[34] Murakami M, Nakagawasai O, Yanai K, Nunoki K, Tan-No K, Tadano T, Iijima T (2007). Modified behavioral characteristics following ablation of the voltage-dependent calcium channel beta3 subunit. Brain Res.

[35] Bidaud I, Mezghrani A, Swayne LA, Monteil A, Lory P (2006). Voltage-gated calcium channels in genetic diseases. Biochim Biophys Acta.

[36] Rea SL, Majcher V, Searle MS, Layfield R (2014). SQSTM1 mutations--bridging Paget disease of bone and ALS/FTLD. Exp Cell Res.

[37] Franks TM, Singh G, Lykke-Andersen J (2010). Upf1 ATPase-dependent mRNP disassembly is required for completion of nonsense- mediated mRNA decay. Cell.

[38] Barmada SJ, Ju S, Arjun A, Batarse A, Archbold HC, Peisach D, Li X, Zhang Y, Tank EM, Qiu H (2015). Amelioration of toxicity in neuronal models of amyotrophic lateral sclerosis by hUPF1. Proc Natl Acad Sci U S A.

[39] Jackson KL, Dayton RD, Orchard EA, Ju S, Ringe D, Petsko GA, Maquat LE, Klein RL (2015). Preservation of forelimb function by UPF1 gene therapy in a rat model of TDP-43-induced motor paralysis. Gene Ther.

[40] Guarguaglini G, Duncan PI, Stierhof YD, Holmstrom T, Duensing S, Nigg EA (2005). The forkhead-associated domain protein Cep170 interacts with Polo-like kinase 1 and serves as a marker for mature centrioles. Mol Biol Cell.

[41] Insolera R, Shao W, Airik R, Hildebrandt F, Shi SH (2014). SDCCAG8 regulates pericentriolar material recruitment and neuronal migration in the developing cortex. Neuron.

[42] Airik R, Slaats GG, Guo Z, Weiss AC, Khan N, Ghosh A, Hurd TW, Bekker-Jensen S, Schroder JM, Elledge SJ (2014). Renal-retinal ciliopathy gene Sdccag8 regulates DNA damage response signaling. J Am Soc Nephrol.

[43] Nagamani SC, Erez A, Bay C, Pettigrew A, Lalani SR, Herman K, Graham BH, Nowaczyk MJ, Proud M, Craigen WJ (2012). Delineation of a deletion region critical for corpus callosal abnormalities in chromosome 1q43-q44. Eur J Hum Genet.

[44] Perlman SJ, Kulkarni S, Manwaring L, Shinawi M (2013). Haploinsufficiency of ZNF238 is associated with corpus callosum abnormalities in 1q44 deletions. Am J Med Genet A.

[45] McKinnon C, Tabrizi SJ (2014). The ubiquitin-proteasome system in neurodegeneration. Antioxid Redox Signal.

[46] Gwizdek C, Casse F, Martin S (2013). Protein sumoylation in brain development, neuronal morphology and spinogenesis. Neuromolecular Med.

[47] Moreno-De-Luca A, Helmers SL, Mao H, Burns TG, Melton AMA, Schmidt KR, Fernhoff PM, Ledbetter DH, Martin CL (2011). Adaptor protein complex-4 (AP-4) deficiency causes a novel autosomal recessive cerebral palsy syndrome with microcephaly and intellectual disability. J Med Genet.

[48] Abou Jamra R, Philippe O, Raas-Rothschild A, Eck SH, Graf E, Buchert R, Borck G, Ekici A, Brockschmidt FF, Nothen MM (2011). Adaptor protein complex 4 deficiency causes severe autosomal-recessive intellectual disability, progressive spastic paraplegia, shy character, and short stature. Am J Hum Genet.

[49] Vanoye CG, Gurnett CA, Holland KD, George AL, Kearney JA (2014). Novel SCN3A variants associated with focal epilepsy in children. Neurobiol Dis.

[50] Kawaji H, Severin J, Lizio M, Waterhouse A, Katayama S, Irvine KM, Hume DA, Forrest AR, Suzuki H, Carninci P (2009). The FANTOM web resource: from mammalian transcriptional landscape to its dynamic regulation. Genome Biol.

[51] Khurana E, Fu Y, Colonna V, Mu XJ, Kang HM, Lappalainen T, Sboner A, Lochovsky L, Chen J, Harmanci A (2013). Integrative annotation of variants from 1092 humans: application to cancer genomics. Science.

[52] Firth HV, Wright CF, Study DDD (2011). The Deciphering Developmental Disorders (DDD) study. Dev Med Child Neurol.

[53] Dixon JR, Selvaraj S, Yue F, Kim A, Li Y, Shen Y, Hu M, Liu JS, Ren B (2012). Topological domains in mammalian genomes identified by analysis of chromatin interactions. Nature.

[54] Kleefstra T, Kramer JM, Neveling K, Willemsen MH, Koemans TS, Vissers LE, Wissink-Lindhout W, Fenckova M, van den Akker WM, Kasri NN (2012). Disruption of an EHMT1-associated chromatin-modification module causes intellectual disability. Am J Hum Genet.

[55] Heidari A, Tongsook C, Najafipour R, Musante L, Vasli N, Garshasbi M, Hu H, Mittal K, McNaughton AJ, Sritharan K (2015). Mutations in the histamine N-methyltransferase gene, HNMT, are associated with nonsyndromic autosomal recessive intellectual disability. Hum Mol Genet.

[56] Gibson WT, Hood RL, Zhan SH, Bulman DE, Fejes AP, Moore R, Mungall AJ, Eydoux P, Babul-Hirji R, An J (2012). Mutations in EZH2 cause Weaver syndrome. Am J Hum Genet.

[57] Veltman JA, Brunner HG (2012). De novo mutations in human genetic disease. Nat Rev Genet.

[58] van Bokhoven H (2011). Genetic and epigenetic networks in intellectual disabilities. Annu Rev Genet.

[59] de Pagter MS, van Roosmalen MJ, Baas AF, Renkens I, Duran KJ, van Binsbergen E, Tavakoli-Yaraki M, Hochstenbach R, van der Veken LT, Cuppen E (2015). Chromothripsis in healthy individuals affects multiple protein-coding genes and can result in severe congenital abnormalities in offspring. Am J Hum Genet.

