# Supplementary material for: Comprehensive whole genome sequence analyses yields novel genetic and structural insights for Intellectual Disability
Source: BMC Genomics. 2017 May 24;18:403. doi: 10.1186/s12864-017-3671-0 (PMC5442678; doi:10.1186/s12864-017-3671-0)
Supplement: Supplementary file 1 — Supplementary Methods - Additional details on methods presented succinctly in main text. (DOCX 53 kb) [file 12864_2017_3671_MOESM1_ESM.docx]

**Additional file 1: Supplementary Methods**

**Patients**

Patients were enrolled from the BC Children’s and Women’s Hospital Provincial Medical Genetics Program. All patients presented with ID (moderate to severe) and brain morphological defects visible on an MRI or CT scan. Patients were clinically assessed by board certified Medical Genetics specialist clinicians. All patients had no family history of ID, and all were products of normal pregnancies with no reported teratogenic exposures. For each of the families, saliva samples were collected (DNA Genotek®) from child, father and mother.

**DNA Extraction from Saliva**

2ml of saliva collected from the Oragene Saliva Collection Kit (OG-500, OG-575). Genomic DNA was extracted following DNA Genotek’s (DNAgenotek®) protocol for purification of whole sample. Briefly, the sample was transferred to a 15ml polypropylene tube and 4% sample volume of PT-L2P (prepIT™•L2P) was added to it. After vortexing and incubating, DNA was precipitated by the addition of 95% ethanol and inversion mixing. After a second incubation, the sample was centrifuged, the supernatant was then discarded and the DNA pellet washed and let stand at room temperature for one minute with 70% ethanol. Then ethanol was removed, and sample re-suspended in TE buffer and incubated in a 50⁰C water incubator for one hour to ensure complete rehydration of DNA. Final DNA concentration was quantified by absorbance method using the NanoDrop Spectrophotometer (ND-1000).

**Whole genome library construction and sequencing**

Paired-end whole genome sequencing (WGS) libraries were prepared from 2µg of genomic DNA using the Illumina’s PCR-free protocol, based on the TruSeq DNA Sample prep kit (Illumina Catalogue Number FC-121-1002). Briefly, 2µg of genomic DNA was fragmented using sonication in a 96-well format. DNA was subjected to end repair, and then size selected using AMPure XP beads to yield DNA fragments of median sizes ranging from 300-400 bp in length. After 3’ end A-tailing, full-length TruSeq adapters were ligated to fragments. Impurities and excess reagents were removed using AMPure XP beads. Library quality was assessed by running an aliquot of PCR-amplified (primers used were AATGATACGGCGACCACCGACACTC and CAAGCAGAAGACGGCATACGAGAT) library DNA on an Agilent 2100 Bioanalyzer DNA1000 chip or a Caliper GX DNA1000 chip. The final PCR-free library concentration was quantified using the KAPA qPCR library Quantification kit (KK4824) prior to generating 100bp paired-end reads on the IlluminaHiSeq 2500 platform using v3 chemistry. A minimum of three lanes of sequencing was performed for each sample to reach the desired haploid sequence coverage. Average coverage for all 24 samples was 34.33X (minimum, median, and maximum coverage of 27.32X, 33.62X and 39.35X, respectively).

**Whole genome sequence data alignment**

Following the removal of reads that failed Chastity filtering [^1^](#_ENREF_1). Raw reads were aligned to the human reference genome GRCh37-lite/hg19a using the Burrows-Wheeler Aligner (BWA; version 0.5.7)[^2^](#_ENREF_2). BAM files were sorted using SAMtools (version 0.1.13)[^2^](#_ENREF_2) and reads were merged and marked for duplicates using Picard MarkDuplicates.jar (version 1.71).

**Single nucleotide variants (SNV) prioritization**

Putative SNVs were identified using SAMtools mpileup version 0.1.17 run on each sample separately. *De novo* variants were selected by intersecting the child’s SNVs with that of each parent, and selecting variants only present in the child and not in either parent, using BEDtools[^10^](#_ENREF_10) intersectBED (version 2.17.0). Final VCF files were annotated using wANNOVAR[^11^](#_ENREF_11).

**Coding SNV analysis.**

For variants in the coding region, we selected *de novo* missense, nonsense and splicing variants. We next selected for rarity removing variants with MAF of >0.01 in 1000G, NHLBI-ESP, Exome Variant Server, Exome Aggregation Consortium (ExAC) and in-house databases of >7430 exomes, and >3000 genomes. We also removed variants in dbSNP135 (excluding disease-associated variants). Next, we prioritized the remaining variants by pathway using three different pathway analyses programs – IPA (
QIAGEN’s Ingenuity® Pathway Analysis), DAVID [^12^](#_ENREF_12) and Panther [^13^](#_ENREF_13), and selected for genes enriched in pathways that were involved in brain development and function. We then annotated pathogenicity predictions for each of the variant that passed our gene-based pathway filter, screening them by SIFT [^14^](#_ENREF_14), PolyPhen[^15^](#_ENREF_15), LRT[^16^](#_ENREF_16) and MutationTaster[^17^](#_ENREF_17) as well as looking at conservation scores using PhyloP[^18^](#_ENREF_18). Variants that were called as highly conserved by PhyloP and were predicted to be damaging by at least one pathogenicity prediction software, were selected for verification by Sanger sequencing in the child, mother and father.

**Non-coding region analyses – *De Novo* Variants in Potential Regulatory Regions (DVPRR)**

For SNVs found in non-coding regions, we applied an additional filtering step to identify *de novo* SNVs that had mapping qualities greater than 30 and a minimum coverage of 10 reads. We also excluded SNVs in regions where the read coverage was greater than 100 to remove SNVs found in repeat regions of the genome where a high read coverage often resulted due to ambiguous mapping of reads. Lastly, we removed SNPs based on the dbSNP database (version 135). We intersected our high-confidence SNVs with several categories of non-coding regulatory regions based on public resources and published literature: (1) Transcription factor binding sites annotated by the ENCODE consortium[^12^](#_ENREF_12) based on ChIP-Seq data that targeted 161 transcription factors [A BED file (version V3) containing 4,380,444 binding site locations was downloaded from the UCSC database http://hgdownload.cse.ucsc.edu/goldenpath/hg19/encodeDCC/wgEncodeRegTfbsClustered/].(2) Putative promoter regions defined as 500bp-upstream of the transcription start site (TSS): We considered 205,537 TSS based on the EnsEMBL gene and transcript model (version 69). (3) 43,011 enhancer regions annotated by the FANTOM consortium: We downloaded the pre-defined enhancer sets and TSS-enhancer association tracks from http://enhancer.binf.ku.dk/Welcome.html in May 2014. (4) “Ultra-conserved” regions i.e. genomic regions under purifying selection, identified by Khurana et al [^13^](#_ENREF_13). 338,179 genomic segments annotated as “sensitive” and “ultrasensitive” were considered. (5) 5’ and 3’ Untranslated regions (UTR) based on the EnsEMBL gene and transcript model (version 69). (6) Topological domains (TAD) from human ES cell lines identified by Dixon et al [^14^](#_ENREF_14).

To identify SNVs in the regulatory regions of ID pathology-related genes, we obtained the list of 995 known pathogenic genes from the Deciphering Developmental Disabilities (DDD) study[^15^](#_ENREF_15).

To identify SNVs in the regulatory regions of ubiquitin proteolysis pathway (UPP) genes, we obtained the list of 137 genes in June 2014 from KEGG belonging to the UPP category. We then selected SNVs located to our regulatory regions and also located to the DDD genes as well as to the UPP genes. For gene regulatory elements not directly associated with a gene, we chose a window of 1Mb to within the gene of interest in the case of enhancer elements, and a window of 5kb to the gene of interest for ultrasensitive regions. SNVs that located within these bounderies were further intersected with TADs to ensure the SNV and gene it is predicted to affect lie within the same TAD.

For CNVs found in non-coding regions, we first selected high-confidence *de novo* CNVs. High confidence *de novo* CNVs were defined as those that were found by two or more CNV detection algorithms. Where the breakpoints called by the CNV detection softwares differed, the common region that was called by both platforms was chosen. These variant datasets were then intersected with five different non-coding sequence annotation databases, and thereafter with the DDD and UPP gene sets, as for the SNVs in DVPRR analysis above.

**Analyses of mutation burden in secondary cohorts, bootstrap analysis and pathway enrichment study**

WES data was downloaded from the UK10K project for 2081 patients with neurofunctional phenotypes and from the 1000G project on 2535 normal individuals (September 2014). We obtained a list of SNVs for each of these patients from their corresponding exome sequence.vcf files, and only considered potentially damaging SNVs (PDSs) (annotated variant categories: coding_sequence_variant, frameshift_variant, incomplete_terminal_codon_variant, inframe_deletion, inframe_insertion, initiator_codon_variant, mature_miRNA_variant, splice_acceptor_variant, splice_donor_variant, stop_gained, stop_lost) for downstream analyses. Figure S2 gives a per patient PDS mutation load. Incidence for PDSs in candidate genes in the positive and negative control cohort as well as bootstrap analysis was carried out on this dataset. A gene was considered variant (‘variant gene’) in a patient if there was ≥1 SNV called in it. A gene-wise variant frequency was calculated across all 2081 cases. We then generated three sampling distributions to infer the frequency of variants. The distributions were based on the 1) mean and 2) median of variant frequencies of 6 randomly sampled genes. 1000 iterations were run to generate each distribution. We compared the mean and median variant frequencies in our six candidate genes to the random distribution.

To ascertain which functional pathways, variant genes in the UK10K cohort were involved in, we performed KEGG pathway enrichment analyses. KEGG pathway information was obtained in June 2014. 467 pathways (55 categories) in total were obtained. We considered pathway that had variant genes in ≥ 50% of cases. For each gene list of interest, we determined extent of the overlap of the list with each KEGG pathway. Significantly enriched pathways were those with Fisher’s Exact Test p-values <0.05. We also repeated this analysis with each KEGG pathway category.

**Copy number variant and structural variant analyses**

All CNVs and SVs identified by the following methods were subsequently also manually assessed using IGV for selection for verification;

FreeC: Control FREE Copy number caller (FREEC) version 6.3[^16^](#_ENREF_16)^,^[^17^](#_ENREF_17) computes and normalizes the number of sequencing reads per equal sized segment of the genome to determine inherited and *de novo* CNVs. The CNV calling pipeline was as follows: (1) reads were re-aligned to the genome using Burrows-Wheeler Aligner (BWA) version 0.5.7 as described above. (2) SAMtools version 0.1.18 [^2^](#_ENREF_2) was used to filter sequencing reads so that all that remained passed chastity and aligned uniquely to the genome (samtools view -b -F 0x0604) and it was also used to generate the appropriate input file format for FREEC (samtools mpileup -C 50 -A -B -Q 0 -q 0 -f); (3) FREEC called CNVs with the default parameters and we specified window=1,000, unique match=TRUE, gem mappability file=out100m1_hg19.gem, ploidy=2, and SNP file=hg19_snp137.SingleDiNucl.1based.txt; (4) bash scripting filtered out CNVs with a percentage of uncertainty of the predicted genotype of greater than five 5 as well as those not on autosomes 1 through 22; (5) CNV regions were considered candidate *de novo* if there was no overlap with either the Mother’s or the Father’s CNVs as determined with BEDTools2 version 2.19.1 subtractBed[^3^](#_ENREF_3). (6) candidate *de novo* CNV regions that contained Ensembl version 75 protein coding genes [^18^](#_ENREF_18)were determined to be of interest with BEDTools2 intersectBed; (7) visual *in silico* validation in Integrative Genomics Browser 2.2 [^19^](#_ENREF_19) required that there was no support for the candidate *de novo* CNV region of interest in either the Mother’s or the Father’s genomes.

CNASeq: CNASeq is an in-house copy number variants (CNV) analysis algorithm. CNAseq calls CNVs by using a Hidden Markov Model approach to segment the genome into regions of consistent copy number[^20^](#_ENREF_20). The segments are based on read counts in genomic bins in each sample. A read-depth of 200 reads was set to define a bin. *De novo* CNVs were selected by identifying CNVs present in the child that were not present in either parent using BEDtools intersectBed (version 2.17.0) and setting a mutual overlap exclusion threshold of 70%.

Large scale structural variant analyses by ABySS and DELLY: Large scale structural variant analysis was performed on each trio data set using two different algorithms. A *de novo* assembly method using ABySS (v1.3.4) and trans-AbySS (v1.4.8) [^21^](#_ENREF_21) and a reference based alignment method DELLY (v0.6.1) [^22^](#_ENREF_22). In addition, ABySS and trans-ABySS were used to identify small scale insertion and deletion events of less than 100bp. For the assembly based method each library was assembled using a multi k-mer approach with the resulting contigs analyzed by alignment to the human reference (GRCh37). Any putative aberrant events compared to the reference which were identified in the child were then screened against both parents to give events specific to the child. Manual review of child specific small scale events was done using the Integrative Genomic Viewer (IGV), comparing read alignments to the reference for the child, mother and father. Large scale events were also identified using DELLY. All events identified in the child were compared against both parents to produce a list of child specific events. Events passed threshold if they had a minimum of 1 split read across the breakpoint, 4 reads spanning the breakpoint and predicted an event of greater than 1kb in size.

Filtration of translocations identified by ABySS: ABySS generated 100bp contigs that spanned the translocation were BLATed versus the human genome browser. Translocations where the contig BLATed back to only the chromosome identified by ABySS, and with a BLAT score > 40, and having above 4 spanning and flanking reads were selected for Sanger verification.

**Availability of supporting data**

The data supporting the results of this article is available in the European Genome-Phenome Archive database (https://www.ebi.ac.uk/ega/home; accession number EGAS00001001386).

**URLs**

UK10K project = www.uk10k.org

1000G project = www.1000genomes.org

KEGG = www.genome.jp/kegg/pathway.html

FANTOM consortium = www.fantom.gsc.riken.jp

DGV: Database of Genomic Variants DGV = www.dgv.tcag.ca

STRING = www.string-db.org

OMIM = www.omim.org

dbSNP135 = www.ncbi.nlm.nih.gov.SNP/

NHLBI-ESP = www.evs.gs.washington.edu

Picard = www.broadinstitute.github.io/picard

GRCh37-lite/hg19a = http://www.bcgsc.ca/downloads/genomes/9606/hg19/1000genomes/bwa_ind/genome

**References**

1. Kircher M, Heyn P, Kelso J. Addressing challenges in the production and analysis of illumina sequencing data. *BMC Genomics.* 2011;12:382.

2. Li H, Handsaker B, Wysoker A, et al. The Sequence Alignment/Map format and SAMtools. *Bioinformatics.* Aug 15 2009;25(16):2078-2079.

3. Quinlan AR, Hall IM. BEDTools: a flexible suite of utilities for comparing genomic features. *Bioinformatics.* Mar 15 2010;26(6):841-842.

4. Chang X, Wang K. wANNOVAR: annotating genetic variants for personal genomes via the web. *J Med Genet.* Jul 2012;49(7):433-436.

5. Huang DW, Sherman BT, Tan Q, et al. The DAVID Gene Functional Classification Tool: a novel biological module-centric algorithm to functionally analyze large gene lists. *Genome biology.* 2007;8(9):R183.

6. Thomas PD, Campbell MJ, Kejariwal A, et al. PANTHER: a library of protein families and subfamilies indexed by function. *Genome Res.* Sep 2003;13(9):2129-2141.

7. Kumar P, Henikoff S, Ng PC. Predicting the effects of coding non-synonymous variants on protein function using the SIFT algorithm. *Nat Protoc.* 2009;4(7):1073-1081.

8. Ramensky V, Bork P, Sunyaev S. Human non-synonymous SNPs: server and survey. *Nucleic Acids Res.* Sep 1 2002;30(17):3894-3900.

9. Chun S, Fay JC. Identification of deleterious mutations within three human genomes. *Genome Res.* Sep 2009;19(9):1553-1561.

10. Schwarz JM, Rodelsperger C, Schuelke M, Seelow D. MutationTaster evaluates disease-causing potential of sequence alterations. *Nature methods.* Aug 2010;7(8):575-576.

11. Pollard KS, Hubisz MJ, Rosenbloom KR, Siepel A. Detection of nonneutral substitution rates on mammalian phylogenies. *Genome Res.* Jan 2010;20(1):110-121.

12. Consortium EP. An integrated encyclopedia of DNA elements in the human genome. *Nature.* Sep 6 2012;489(7414):57-74.

13. Khurana E, Fu Y, Colonna V, et al. Integrative annotation of variants from 1092 humans: application to cancer genomics. *Science.* Oct 4 2013;342(6154):1235587.

14. Dixon JR, Selvaraj S, Yue F, et al. Topological domains in mammalian genomes identified by analysis of chromatin interactions. *Nature.* May 17 2012;485(7398):376-380.

15. Firth HV, Wright CF, Study DDD. The Deciphering Developmental Disorders (DDD) study. *Developmental medicine and child neurology.* Aug 2011;53(8):702-703.

16. Boeva V, Zinovyev A, Bleakley K, et al. Control-free calling of copy number alterations in deep-sequencing data using GC-content normalization. *Bioinformatics.* Jan 15 2011;27(2):268-269.

17. Boeva V, Popova T, Bleakley K, et al. Control-FREEC: a tool for assessing copy number and allelic content using next-generation sequencing data. *Bioinformatics.* Feb 1 2012;28(3):423-425.

18. Cunningham F, Amode MR, Barrell D, et al. Ensembl 2015. *Nucleic Acids Res.* Jan 2015;43(Database issue):D662-669.

19. Thorvaldsdottir H, Robinson JT, Mesirov JP. Integrative Genomics Viewer (IGV): high-performance genomics data visualization and exploration. *Briefings in bioinformatics.* Mar 2013;14(2):178-192.

20. Jones SJ, Laskin J, Li YY, et al. Evolution of an adenocarcinoma in response to selection by targeted kinase inhibitors. *Genome biology.* 2010;11(8):R82.

21. Robertson G, Schein J, Chiu R, et al. De novo assembly and analysis of RNA-seq data. *Nature methods.* Nov 2010;7(11):909-912.

22. Rausch T, Zichner T, Schlattl A, Stutz AM, Benes V, Korbel JO. DELLY: structural variant discovery by integrated paired-end and split-read analysis. *Bioinformatics.* Sep 15 2012;28(18):i333-i339.
