# Supplementary material for: Comprehensive whole genome sequence analyses yields novel genetic and structural insights for Intellectual Disability
Source: BMC Genomics. 2017 May 24;18:403. doi: 10.1186/s12864-017-3671-0 (PMC5442678; doi:10.1186/s12864-017-3671-0)
Supplement: Supplementary file 5 — Figure S1. IGV image and Sanger verification trace files for indel in ARID1B and missense variation in UPF1. Figure S2. UK10K mutation load – counts as per variant annotation type on one patient. Figure S3. Histogram of mutation burden per patient in the UK10K cohort. Figure S4. Pathway interactions showing convergence onto UPP pathway. Figure S5. Plots for CNV distribution for two chromosomes as called by CNAseq. (DOCX 1972 kb) [file 12864_2017_3671_MOESM5_ESM.docx]

**A**


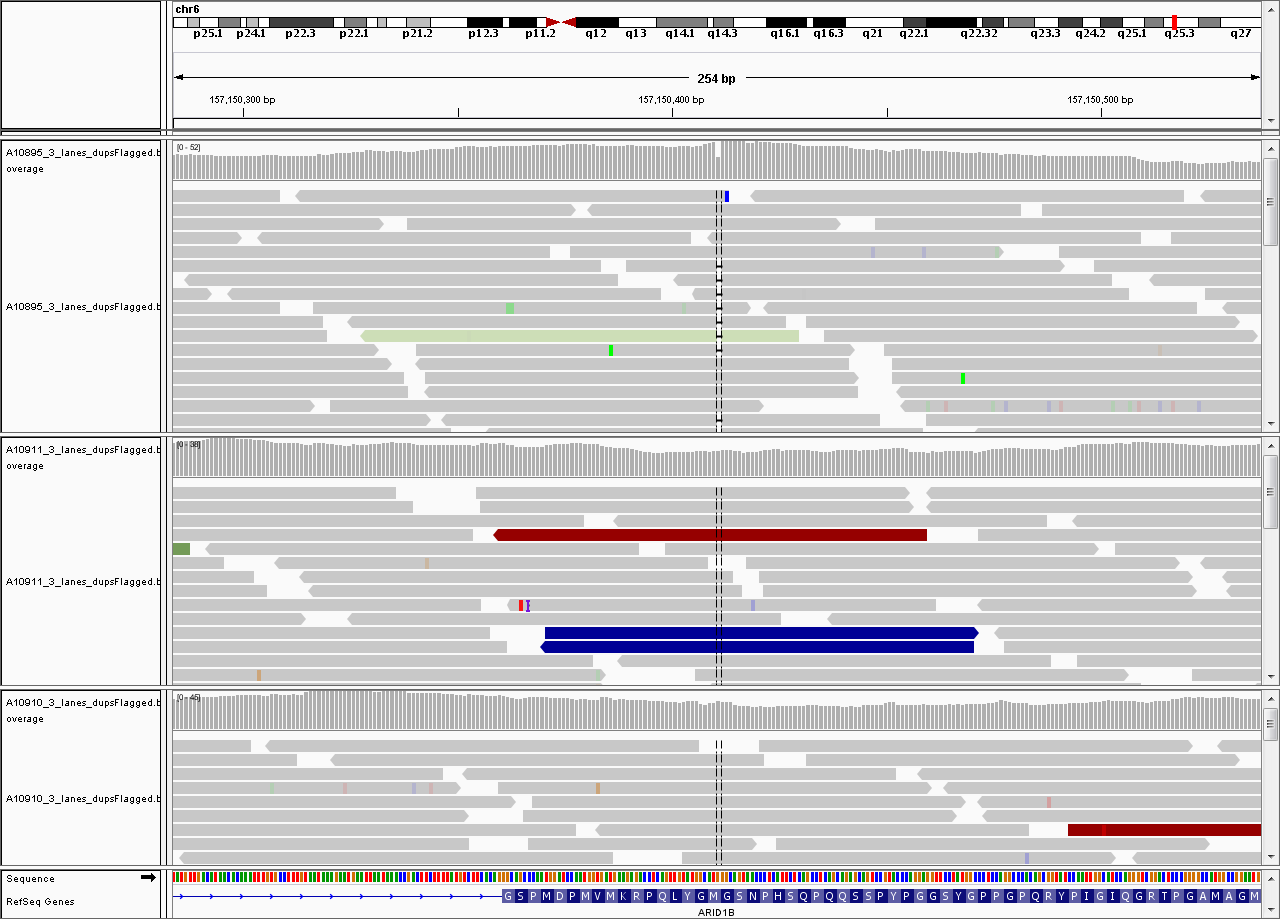

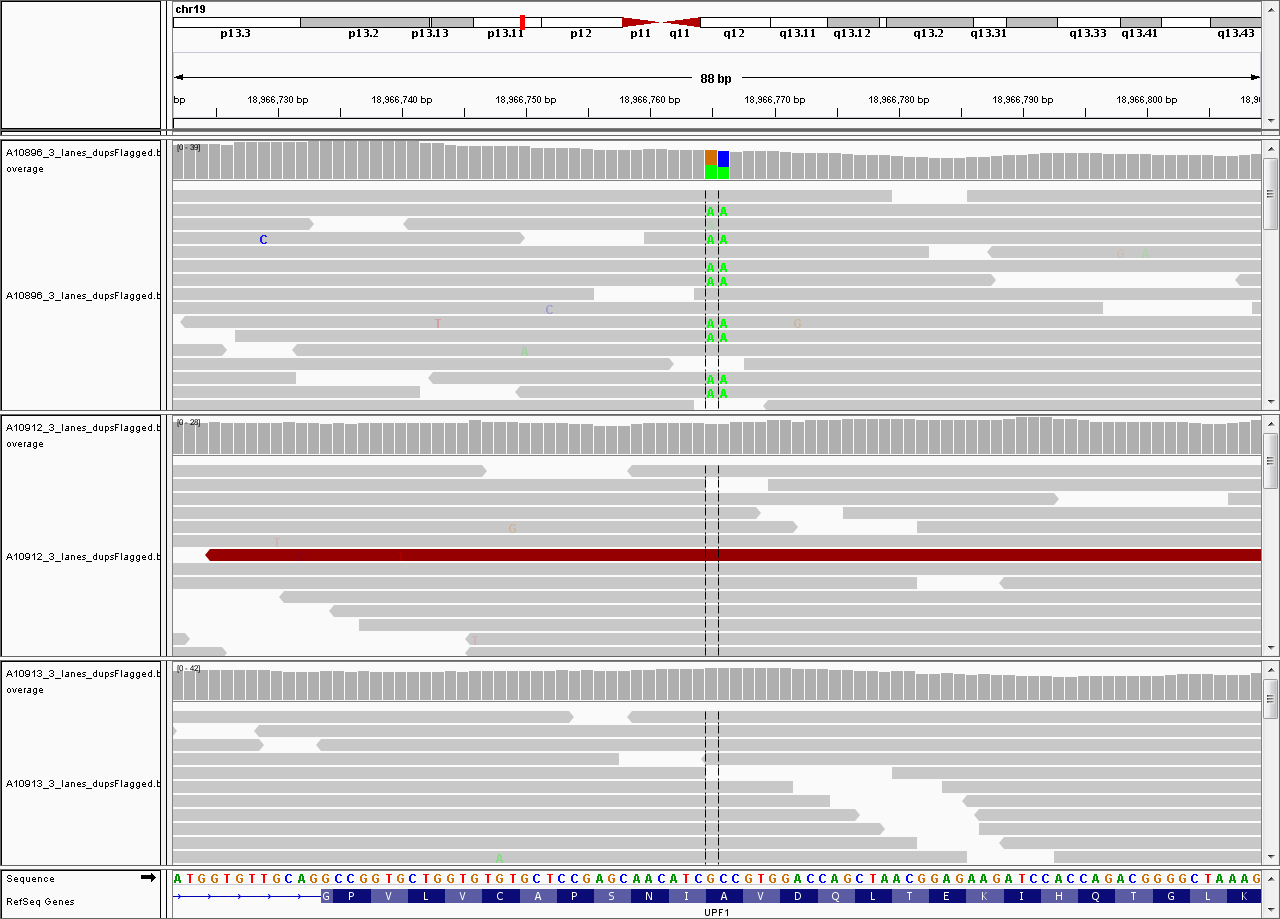

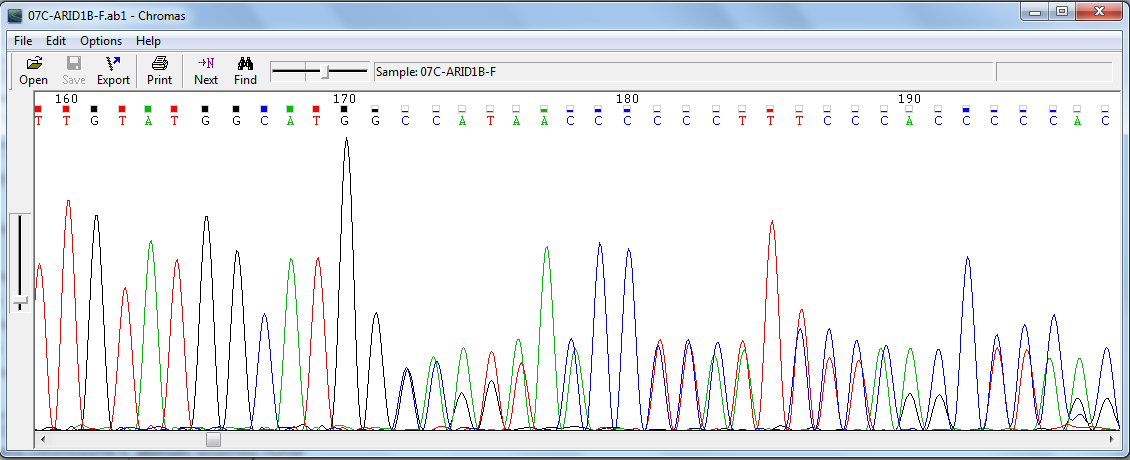

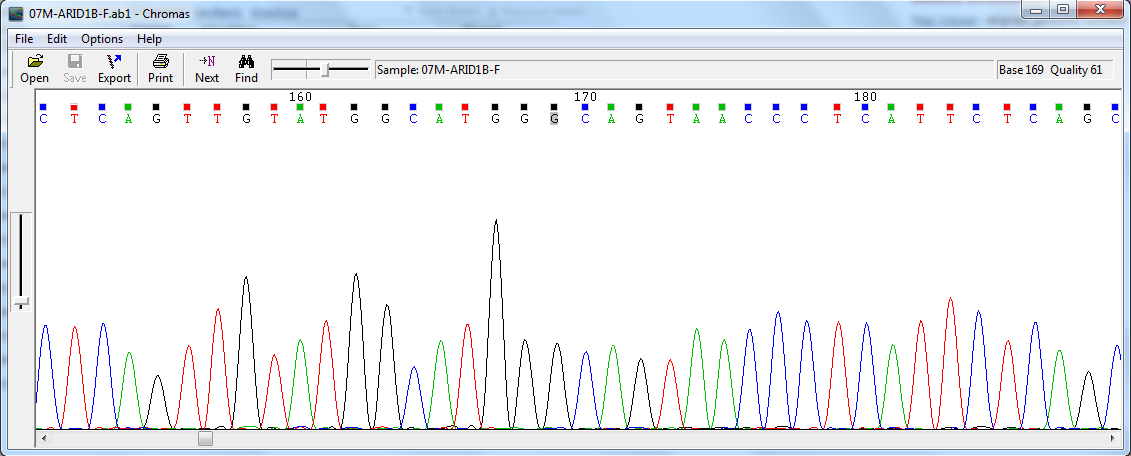

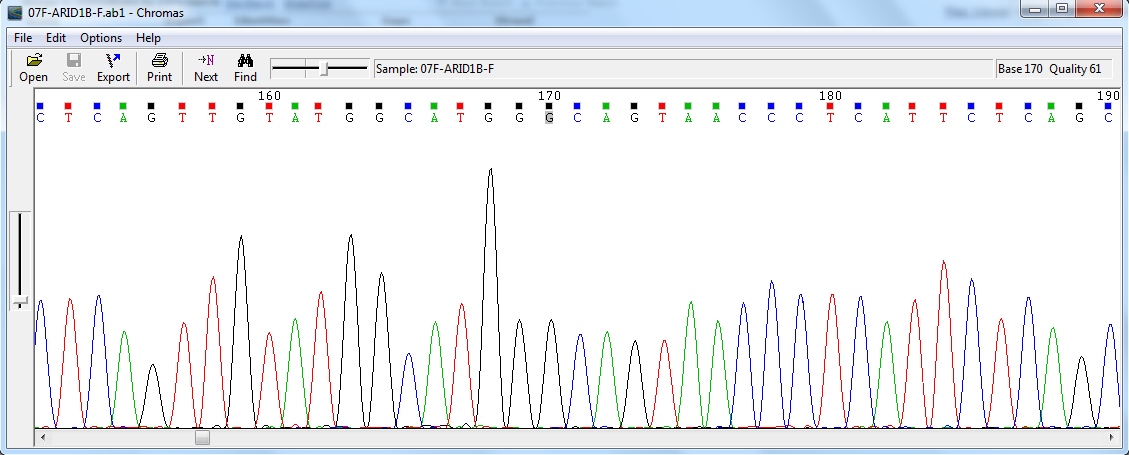

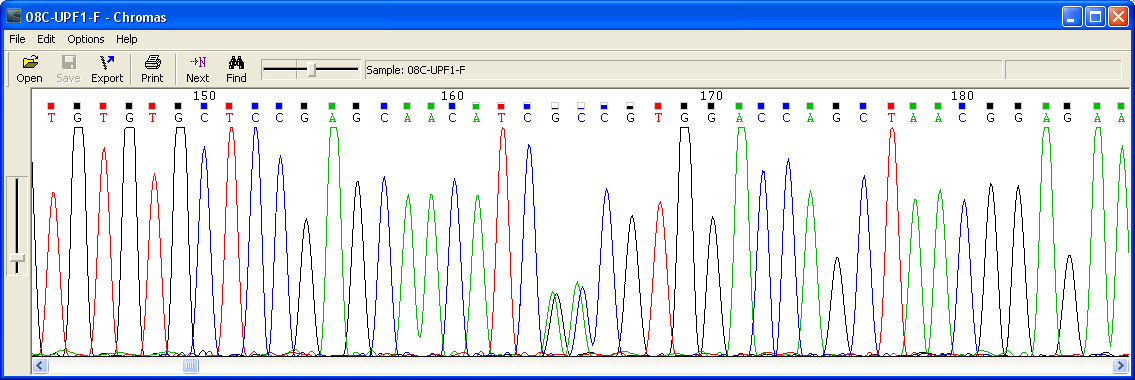

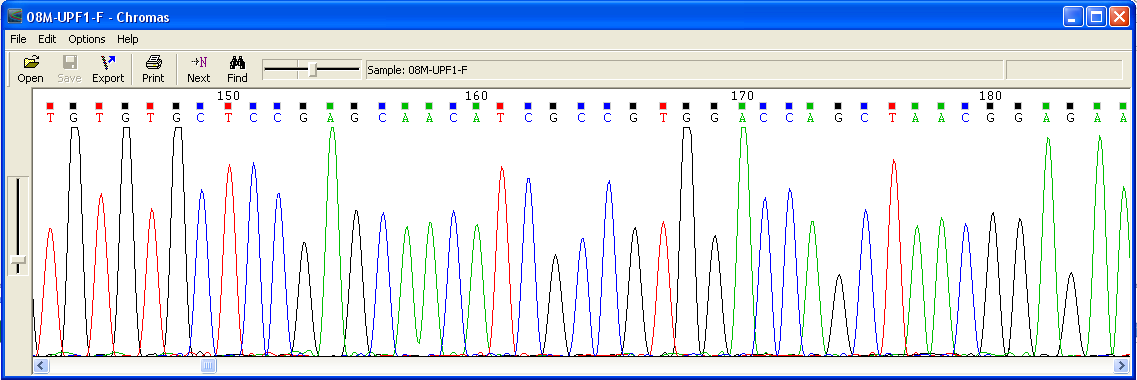

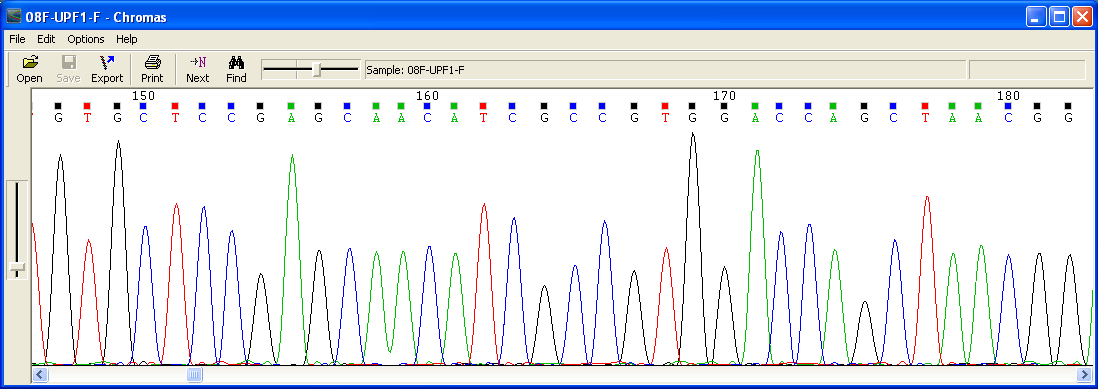


**B**

UPF1

ARID1B

Father

Mother

Child

Father

Father

Father

Mother

Mother

Mother

Child

Child

Child

Figure S1. IGV image and Sanger verification trace files for indel in *ARID1B* and missense variation in *UPF1*. IGV images shows panels for child’s, mother’s and father’s WGS data ordered from top to bottom. Sanger sequnce trace files are shown for child, mother and father ordered from top to bottom.


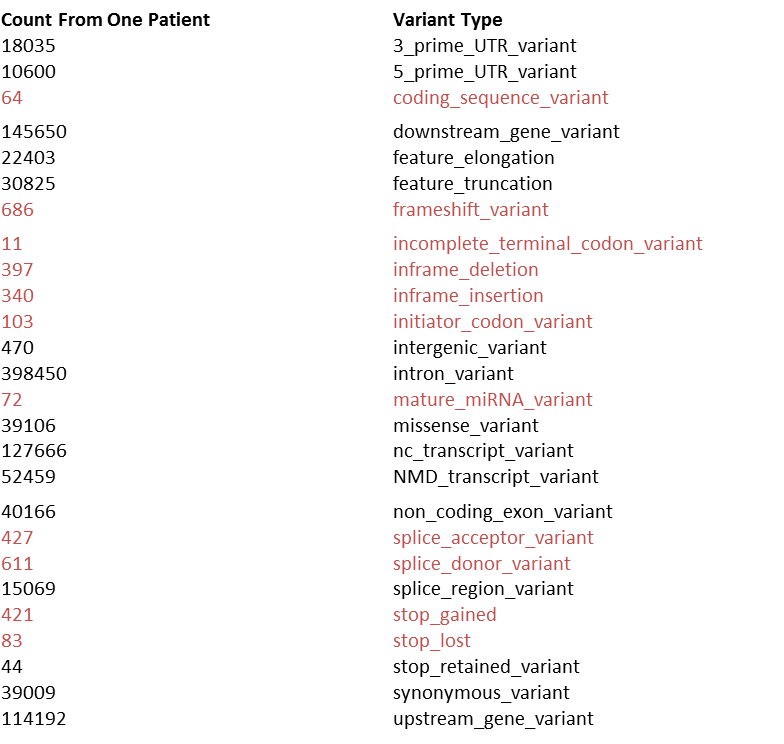


Figure S2. UK10K mutation load – counts as per variant annotation type on one patient. Annotations in red text were used for analyses

Min. 1st Qu. Median Mean 3rd Qu. Max.

18360 19200 19340 19490 19700 22040

Figure S3. Histogram of mutaiton burden per patient in the UK10K cohort


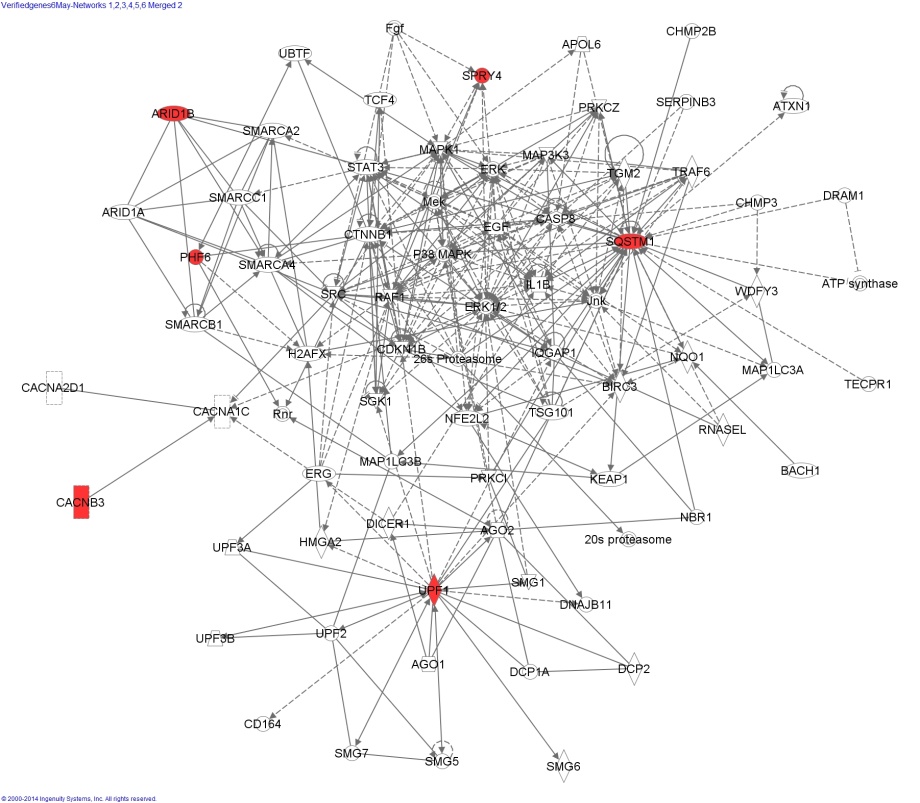

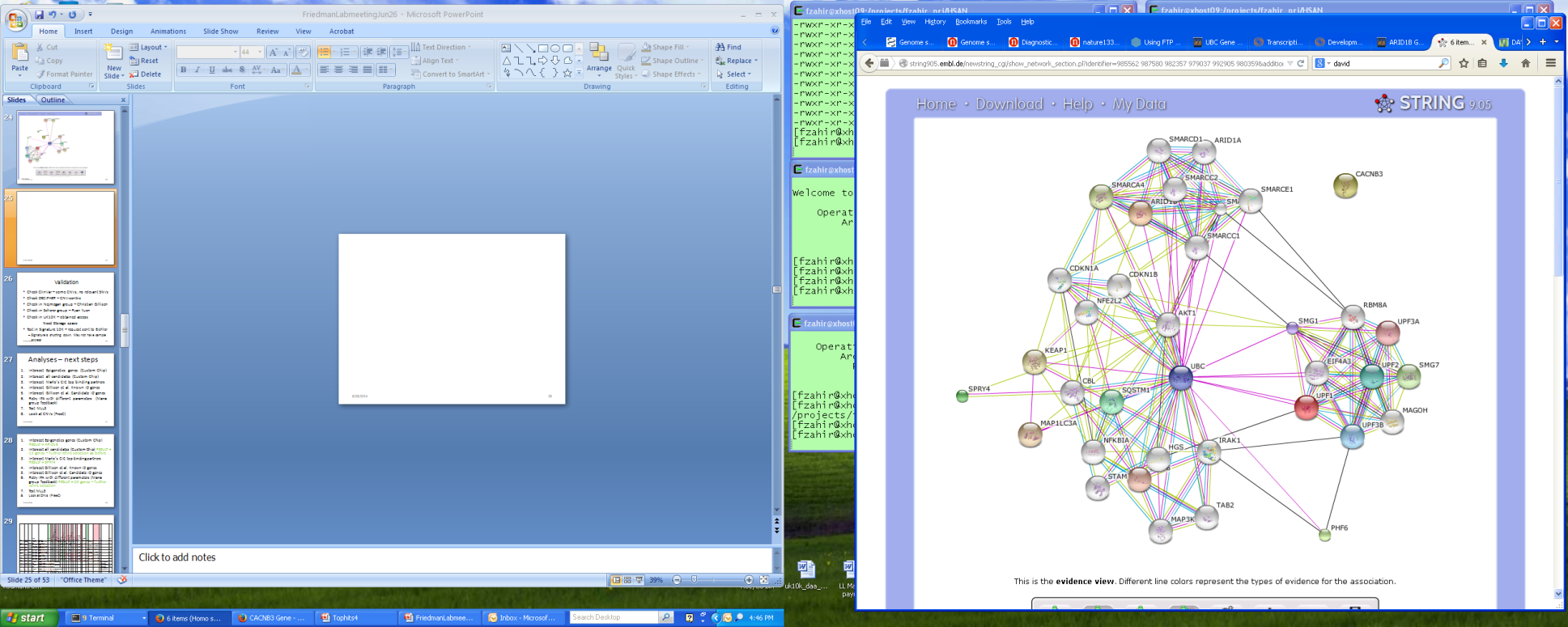


Figure S4. Pathway interactions of 6 candidate genes converge onto UPP pathway: A) IPA result B) STRING.db result


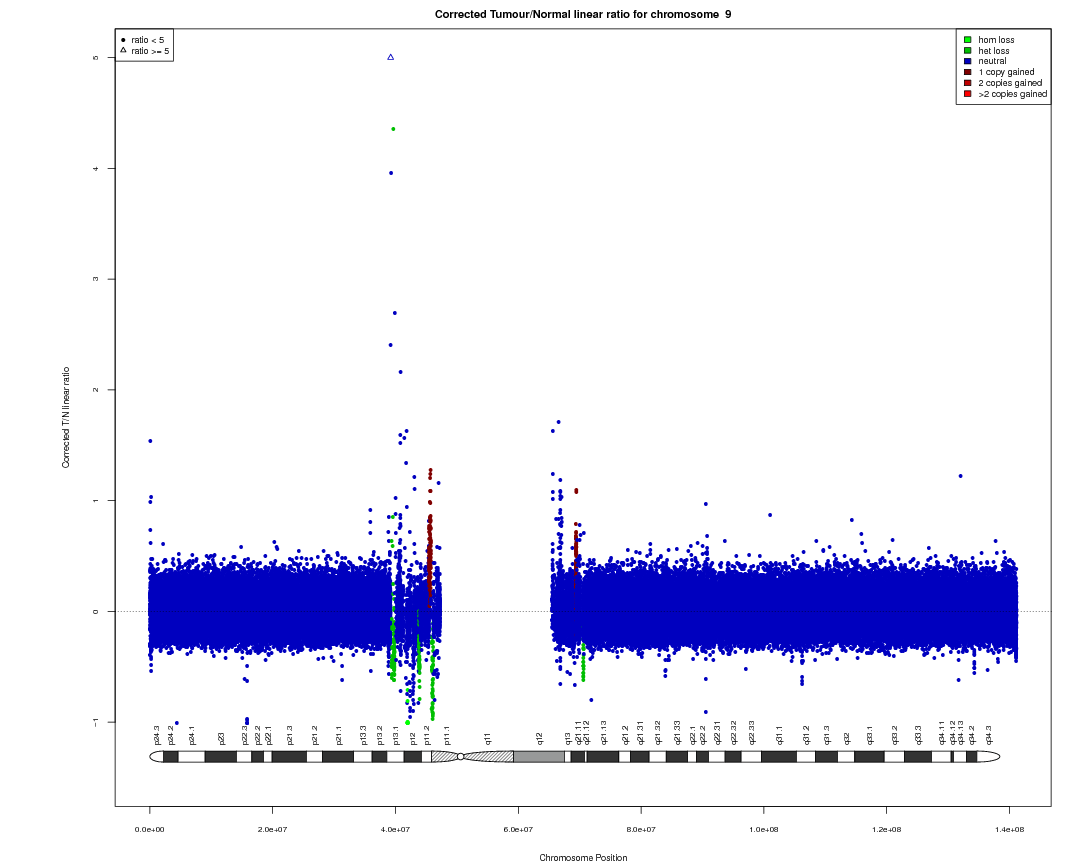

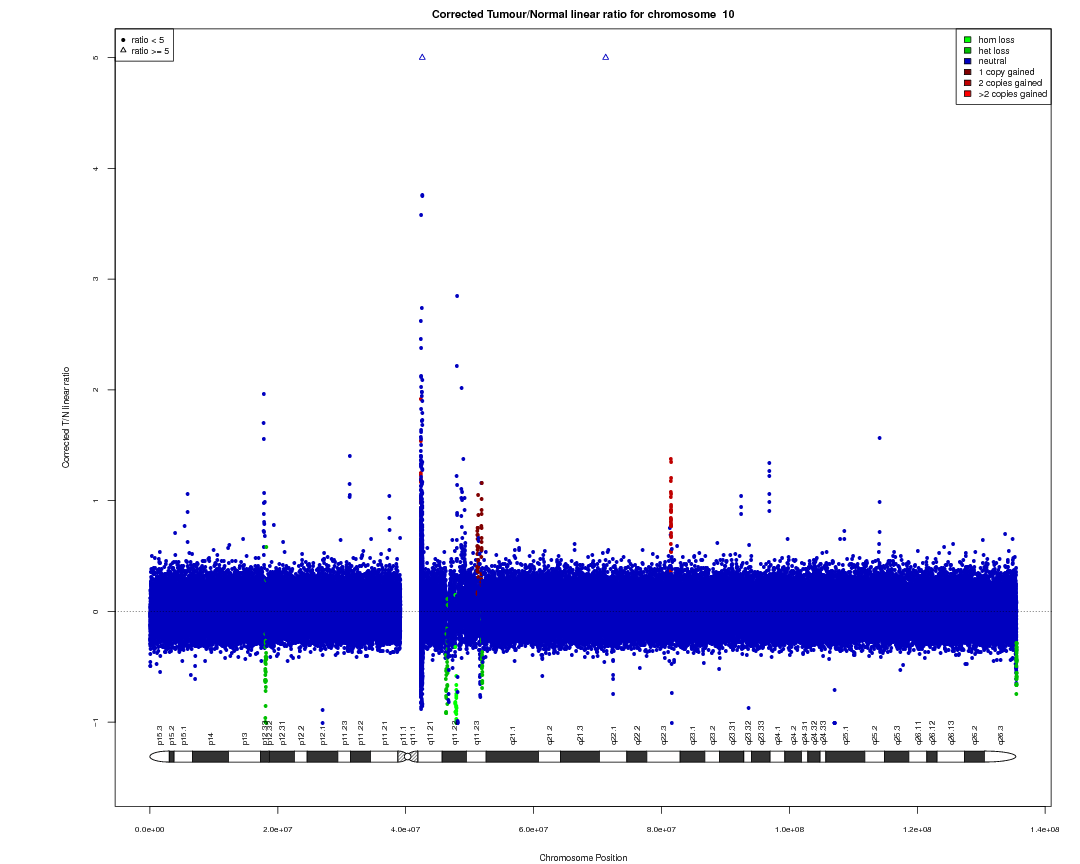


Figure S5. Plots for CNV distribution for two chromosomes as called by CNAseq. Note the preponderance of CNV calls at the centromere and telomere
